# ‘Fengtang’ plum seed waste: Phytochemicals and anti-inflammatory effects *in vivo* and *in vitro*

**DOI:** 10.1016/j.fochx.2026.103793

**Published:** 2026-03-26

**Authors:** Yuanquan Ran, Lu Jin, Furong Ding, Dan Long, Guo Chen, Qiong Hu, Bing Yang, Wenyu Wu, Dongxin Tang, Minyi Tian

**Affiliations:** a Guizhou University of Traditional Chinese Medicine, Guiyang 550000, China; b National & Local Joint Engineering Research Center for the Exploitation of Homology Resources of Southwest Medicine and Food, Guizhou University, Guiyang 550025, China

**Keywords:** ‘Fengtang’ plum, Phytochemicals, Colitis, MAPK, NF-κB

## Abstract

During ‘Fengtang’ plum fruit processing, the seeds as by-products are discarded as waste. Nevertheless, its seed has edible and medicinal value. Thus, we investigated the phytochemical constituents of ‘Fengtang’ plum seed and explored its anti-colitis effects and related mechanisms. ‘Fengtang’ plum seed 70% ethanol extract (EE) and water extract (WE) were found to be abundant in phenolic and flavonoid compounds. Fifty-five phytochemicals identified by UHPLC-Q-Orbitrap-MS analysis included 12 phenols and 8 flavonoids. In LPS-treated RAW264.7 cells, EE inhibited the ROS/MAPK/NF-κB pathway, thereby repressing IL-6, IL-1β, NO, PGE_2_, and TNF-α oversecretion. Additionally, the DSS-induced pathological symptoms of colon mice, including weight loss, colon shortening, and DAI score rise, were considerably alleviated. It also significantly suppressed inflammatory cytokine secretion and reduced ROS generation by increasing CAT and SOD activities and decreasing MDA levels in tissue fluid and serum. Therefore, ‘Fengtang’ plum seed has potential utilization value in the field of functional foods.

## Highlights

• ‘Fengtang’ plum seed waste has intestinal protective effects.
• ‘Fengtang’ plum seed was rich in phenolic and flavonoid compounds.
• It suppressed ROS-mediated activation of MAPK and NF-κB pathways.
• It improved colitis by reducing DSS-induced inflammatory cytokine and ROS levels.
• ‘Fengtang’ plum seed waste has high exploitation potential in functional food field.

## 1 Introduction

Fruits processed industrially into juices, jams, and preserves generate substantial waste and by-products, while the seeds are usually discarded as trash (Liu et al., 2018). Numerous studies have demonstrated that seed waste is abundant in bioactive compounds and possesses a variety of biological activities (Sławińska & Olas, 2022; Sorrenti et al., 2023). To increase the added value of seed waste, researchers are paying more attention to using seed waste as raw materials to develop functional foods with positive health benefits.

Currently, European plum (*Prunus domestica* L.) and Japanese plum (*Prunus salicina* Lindl.) are the most commercially produced plum varieties (Hamdani et al., 2022). According to data from the FAO (Food and Agriculture Organization of the United Nations), the global plum production in 2023 was approximately 12 million tons, of which China accounted for more than half (6.9 million tons) (FAO, 2023). When plum is eaten or processed into tins and preserved fruit, the remaining plum seed (constituting 3–5% of the overall fruit mass) is usually wasted out as trash. However, the plum seed has edible and medicinal value. Plum seed is rich in protein and fatty acids, which have been processed into plant-based protein drinks and edible oil (Gao et al., 2024; VladiÄ et al., 2020). As a traditional Chinese medicinal material, plum seed is included in the Chinese Pharmacopoeia and is commonly utilized to relieve various symptoms associated with colitis, such as bloating, indigestion, constipation, and abdominal pain (China Pharmacopoeia Committee, 2020; Nanjing University of Chinese Medicine, 2006). According to prior studies, plum seed oil contains plenty of unsaturated fatty acids, especially oleic and linoleic acids (Rodríguez-Blázquez et al., 2023). Plum seed oil exhibits antibacterial, anti-inflammatory, and free radical scavenging properties (Fratianni et al., 2021). Besides, plum seed is plentiful in flavonoids and phenolics and has antibacterial, anticancer, and free radical scavenging efficacy (Khallouki et al., 2012; Savić et al., 2016; Younis et al., 2023). ‘Fengtang’ plum is a plum cultivar (*Prunus salicina* Lindl.) in Guizhou, China, and it was authorized by the Crop Variety Approval Committee of Guizhou, China, in 2016 (approval number: *Qianshenguo* 2016002) (Department of Agriculture and Rural Affairs of Guizhou Province, 2016). Due to its rich flavor and crisp taste, ‘Fengtang’ plum is popular among consumers. However, there are currently no reports on the chemical composition and bioactivity of ‘Fengtang’ plum seeds.

Functional foods offer health benefits beyond conventional nutrients (Milner, 2000). A variety of functional foods have been used to maintain intestinal health through antioxidant activity and improvement of intestinal damage (Cencic & Chingwaru, 2010; Mijan & Lim, 2018). Plant-derived natural products, especially phenolics and flavonoids, have been widely demonstrated to eliminate reactive oxygen species (ROS) and applied in the prevention and therapy of disorders associated with excessive ROS production (Huyut et al., 2017; Mbinda & Musangi, 2019). ROS act as central regulators of inflammatory signaling pathways, and excessive production and long-term exposure to ROS can induce oxidative stress and are closely linked to the onset and progression of colitis (Wang et al., 2020a). The course of colitis is accompanied by overproduction of inflammatory factors (Kim et al., 2018). In colitis, the MAPK/NF-κB pathway is crucial for controlling the release of inflammatory factors (Sun et al., 2013). Excessive ROS exacerbates intestinal inflammation by activating the MAPK/NF-κB pathway (Wan et al., 2022). Therefore, inhibition of the ROS/MAPK/NF-κB pathway is an efficacious strategy for treating colitis.

‘Fengtang’ plum seed is often discarded as waste. However, plum seed possesses nutritional and medicinal value and has been utilized to relieve various symptoms associated with colitis. Hence, the purpose of our study is to examine the chemical components and anti-colitis properties of ‘Fengtang’ plum seed, thereby providing a theoretical basis for its application in functional foods.

## 2 Materials and methods

### 2.1 Chemical and reagents

Solarbio Sciences & Technology supplied the dexamethasone (DXM), gallic acid, rutin, Folin-Ciocalteu reagent, lipopolysaccharide (LPS), and DMSO (Beijing, China). Sigma-Aldrich (Germany) supplied the ABTS, DPPH, BHT, MTT, ascorbic acid, and BHT. Beyotime Biotechnology (Shanghai, China) supplied the BCA protein concentration assay kit, nuclear and cytoplasmic protein extraction kit, ROS assay kit, NO detection kit, and RIPA lysis buffer. Abnova Corporation offered the PGE_2_ ELISA kit (Taiwan, China). We acquired the ELISA kits for mouse TNF-α and IL-6 from Lianke Biotech, Co., Ltd. (Hangzhou, China). Antibodies were bought from CST (Danvers, MA, USA). Mesalazine was from Losan Pharma GmbH (Freiburg im Breisgau, BW, Germany). OMEGA Bio-Tec (Norcross, USA) offered the E.Z.N.A.® Total RNA Kit I. RT Easy™ II kit and Real-time PCR Easy™-SYBR Green I kit were provided by Foregene Biotechnology Co., Ltd. (Chengdu, China). Meilun Bio Co., Ltd. (Dalian, China) offered the dextran sulfate sodium (DSS). Mouse IL-1β ELISA kit and the kits of SOD, CAT, and MDA were bought from Raysun Bio Co., Ltd. (Quanzhou, China). Servicebio provided H&E staining kits and general tissue fixatives (Wuhan, China). The 30% H_2_O_2_ was purchased from Chuandong Chemical Co., Ltd. (Chongqing, China). Diprovocim was provided by MCE (Shanghai, China).

### 2.2 Plant material

The ‘Fengtang’ plum used in this study was purchased in September 2021 from the orchard of Zhenning Liuma ‘Fengtang’ plum Development Co., Ltd., Liuma Town, Zhenning County, Anshun City, Guizhou Province, China (25°63′74.08“ N and 105°85’39.07” E). The species was identified as ‘Fengtang’ plum by Professor Guoxiong Hu of Guizhou University (specimen number: PS20210727).

### 2.3 Preparation of ‘Fengtang'plum seed WE and EE

The ‘Fengtang’ plum seeds were washed, air-dried, and then ground using a high-speed grinding machine. The 2 L of ultrapure water were added to a 5 L round-bottom flask containing the 0.5 kg ground material and refluxed for extraction twice, each lasting 2 h. After being combined and filtered, the extracted filtrates were concentrated. Next, a vacuum freeze dryer was used for drying to obtain the ‘Fengtang’ plum seed WE. In the EE preparation, the solvent of the reflux extraction was replaced with 70% ethanol, and EE was prepared following the same steps as above. Finally, samples were weighed to calculate the extraction yield and preserved for further use in a desiccator in brown bottles.

$$\text{The yield}\;\left(\%\right)=\frac{{\text{weight}}_{\left(\text{WE or}\;\mathrm{EE}\right)}}{\;{\text{weight}}_{\text{fresh seeds}}}\times 100\%$$

### 2.4 TPC and TFC test

#### 2.4.1 TPC test

Briefly, 2.5 mL of Folin-Ciocalteu reagent was mixed with 0.5 mL of ‘Fengtang’ plum seed solution (WE or EE), and the mixture was left to incubate in the dark for 4 min. The mixture was then incubated for 1 h in the dark after 2 mL of 7.5% Na_2_CO_3_ solution was added. The standard curve was constructed by applying linear regression to plot absorbance (y-axis) against gallic acid dosage (x-axis) after the absorbance measurement at 760 nm. Next, TPC was computed and represented as mg of the gallic acid equivalent per g of ‘Fengtang’ plum seed extract.

#### 2.4.2 TFC test

Briefly, ‘Fengtang’ plum seed solution (5 mL, WE or EE) was blended with 5% NaNO_2_ solution (0.4 mL). After 6 min of reaction, 10% Al(NO_3_)_3_ solution (0.4 mL) was incorporated and incubated lasting 6 min. Then, we added ultrapure water (0.2 mL) and 4% NaOH solution (4 mL). After 15 min of reaction and recording the absorbance (510 nm), the relationship between absorbance and rutin dosage was plotted by linear regression to establish a standard curve. The TFC of sample was calculated from the standard curve, and phenolic concentration in the extracts is represented as mg of rutin equivalent per g of ‘Fengtang’ plum seed extract.

### 2.5 Free radical scavenging effects of ‘Fengtang’ plum seed WE or EE

The free radical scavenging activities were assessed using DPPH and ABTS assays. Positive controls were ascorbic acid and BHT, with IC_50_ values representing the results.

#### 2.5.1 ABTS free radical scavenging

ABTS (9.6 mg) was dissolved in anhydrous ethanol to prepare the ABTS solution (0.7 mM). K_2_S_2_O_4_ (16.5 mg) was dissolved in pure water to prepare K_2_S_2_O_4_ solution. For the production of the ABTS•^+^ solution, the above two solutions were combined in equal amounts and reacted in dark for 16 h. Three groups were set up in the further experiment, including blank, control, and sample groups. ABTS•^+^ solution (180 mL) and pure water (18 mL) were pipetted into the blank group. The control group was added with the sample solution (18 mL) and pure water (180 mL). Besides, ABTS•^+^ solution (180 mL) and sample solution (18 mL) were pipetted into the sample group. Thereafter, all groups were reacted for 10 min in darkness. At 734 nm, the optical density (OD) was recorded.

$$\text{Radical}-\text{scavenging rate}\;\left(\%\right)=\left[1-\frac{{\mathrm{OD}}_{\text{sample}}-{\mathrm{OD}}_{\text{control}}}{{\mathrm{OD}}_{\text{blank}}}\right]\times 100\%$$

#### 2.5.2 DPPH free radical scavenging

To prepare a 0.08 mM DPPH solution, 9.85 mg DPPH was blended with 25 mL of anhydrous ethanol. Three groups were established, including blank, control, and sample groups. Anhydrous ethanol and DPPH solution (100 μL) were combined in equal proportions in the blank group. The control group consisted of a sample solution (‘Fengtang’ plum seed WE or EE) combined with anhydrous ethanol (100 μL) in equal amounts. Besides, the sample group was received the equal volume (100 μL) of sample solution and DPPH solution. After that, all groups were incubated for 30 min in darkness. At 517 nm, the optical density (OD) was recorded, and the calculation formula is the same as above.

### 2.6 Phytochemical identification of ‘Fengtang’ plum seed extracts

UHPLC-Q-Orbitrap-MS (Thermo Scientific Dionex Ultimate 3000 RSLC) was utilized to examine the phytochemicals of ‘Fengtang’ plum seed extracts. The following were the UHPLC parameters set: injection volume (5 μL), mobile phases (phase A: 0.1% formic acid acetonitrile and phase B: 0.1% formic acid water solution), column temperature (40 °C), flow rate (0.3 μL/min), and column (ACE Ultracore 2.5 Super C_18_, 1.9 μm, 2.1 mm × 100 mm). The following was the gradient elution program: 95% B (0–2 min), 95% ∼ 5% B (2–42 min), 5% B (42–47 min), 95% B (47–47.1 min), and 95% B (47.1–50 min).

The MS data were obtained using the Q-Orbitrap-MS with HESI-II. The following were the HESI-II parameters: S-lens (60), probe heater and capillary temperature (350 and 320 °C), sheath, auxiliary, and sweep gas (35, 10, and 0 arb), and spray voltages (+3.0/−2.5 kV). The following scanning parameters for mass spectrometry were used: stepped NCE (20, 40, and 60 eV), intensity threshold (1.6 e^5^), loop count (3), isolation width (1.5 *m/z*), dynamic exclution (5 s), AGC target (MS^1^: 1 × e^6^, MS^2^: 2 × e^5^), MS^1^ scan range (100–1500 *m/z*), resolution (MS^1^: 70,000, MS^2^: 17,500), and maximum AGC target (8 × e^3^). The mass spectrometry data of phytochemicals were analyzed using the Xcalibur 4.1 program with the mass tolerance threshold setting of 10 ppm. Phytochemical constituents were identified using the mzVault and mzCloud databases and the references (detailed analysis of fragmentation patterns in Supplementary Material).

### 2.7 Detection of *in vitro* anti-inflammatory properties of ‘Fengtang’ plum seed

#### 2.7.1 Cytotoxicity assay

The MTT assay was used to evaluate the cytotoxicity of ‘Fengtang’ plum seed extracts on RAW264.7 cells. To prepare a sample solution, the extracts were dissolved in DMSO and then mixed with culture medium (ensuring that the DMSO content was less than 0.05%). After being inoculated at a density of 2 × 10^5^ cells/mL in a 96-well plate, cells were cultured at 37 °C for 24 h. Then, we replaced the previous culture medium with 200 μL of the sample solution. Next, MTT solution (10 μL, 5 mg/mL) was injected into each well after 24 h of incubation. After 4 h, the supernatant was discarded, and DMSO (150 μL) was injected to each well. The optical density at 490 nm was recorded after shaking the plate for 10 min.

#### 2.7.2 Determination of inflammatory factor levels

The LPS group (model group), DXM group (positive group), control group, and ‘Fengtang’ plum seed extract group were set up. After being injected into 96-well plates, RAW264.7 macrophages (100 μL, 2 × 10^6^ cells/mL) were cultured for 24 h at 37 °C. After discarding the old medium, the positive group received DXM solution (200 μL, 20 μg/mL), whereas the LPS and control groups received medium (200 μL), and WE or EE solution (200 μL) was added to the ‘Fengtang’ plum seed extract group. After 2 h of incubation, the previous culture medium was removed. Immediately after, 200 μL of culture solution was added to the control group, and LPS solution (200 μL, 1 μg/mL) was added to the LPS group. For the positive group, a 200 μL mixture solution with DXM (20 μg/mL) and LPS (1 μg/mL) was injected. Besides, 200 μL of an equal volume of mixed solutions with LPS (1 μg/mL) and ‘Fengtang’ plum seed extracts (62.5, 125, and 250 μg/mL) were added to the ‘Fengtang’ plum seed extract group. After incubation for 24 h, the supernatant was collected, and the NO content was determined using a NO detection kit. Additionally, ELISA kits were used to measure the levels of TNF-α, PGE_2_, IL-6, and IL-1β.

In addition, to further confirm that EE exerts its anti-inflammatory effect through the ROS/MAPK/NF-κB pathway, we used H_2_O_2_ (ROS activator) and diprovocim (MAPK and NF-κB pathway activator) to observe whether they could reverse the inhibitory effect of EE on NO secretion. The LPS group (model group), control group, ‘Fengtang’ plum seed EE group (250 μg/mL), H_2_O_2_ group, and diprovocim group were established. Following the steps described above, cells in the LPS group were treated with LPS (1 μg/mL) alone, while cells in the EE, H_2_O_2_, and diprovocim groups were treated with LPS (1 μg/mL) and ‘Fengtang’ plum seed EE (250 μg/mL). Next, the supernatant from each group was aspirated. Subsequently, 200 μL of fresh medium was added to the LPS, control, and ‘Fengtang’ plum seed EE groups. The H_2_O_2_ group was supplemented with H_2_O_2_ solution (2000 μM, 200 μL), and the diprovocim group was supplemented with diprovocim solution (500 nM, 200 μL). After 12 h of incubation, the supernatant was collected, and NO levels were measured using a NO detection kit.

#### 2.7.3 Morphological observations and determination of ROS levels

The DCFH-DA fluorescent probe was used to measure intracellular ROS scavenging activity. After being plated into 6-well plates, RAW264.7 cells (1 mL, 6 × 10^5^ cells/mL) were incubated for 1 day. Next, different concentrations of EE solutions (1 mL, 62.5, 125, and 250 μg/mL) were used to replace the old culture medium. After pretreating for 2 h and discarding the previous culture medium, 2 mL of mixed solutions with EE (concentration as above) and LPS (1 μg/mL) were added. Following a 24 h culture period, the morphology of RAW264.7 cells was viewed using a microscope. Afterward, the previous medium was thrown away, and serum-free media was used to wash twice the cells. Thereafter, 700 μL DCFH-DA solution was added and reacted for 20 min. After being rinsed with serum-free media, the cells were viewed and photographed using a Leica DMi8 fluorescent microscope.

To verify whether the anti-inflammatory effect of EE is mediated by ROS scavenging, we used H_2_O_2_ (ROS activator) to determine whether it could reverse EE's ROS-scavenging effect. Following the method described above, RAW264.7 cells were treated with EE (250 μg/mL) and LPS. Subsequently, the old culture medium was discarded. H_2_O_2_ solution (2000 μM, 2 mL) was added and incubated for 12 h. Afterward, the cells were stained with 700 μL DCFH-DA solution for 20 min, and images were captured using a Leica DMi8 fluorescence microscope.

#### 2.7.4 qRT-PCR

After treating macrophages with ‘Fengtang’ plum seed EE using the aforementioned method, E.Z.N.A.® total RNA kit I was used to extract total RNA, and an ultraviolet spectrophotometer was used to determine its concentration. Next, the RT Easy™ II kit was utilized to reverse transcribe RNA into cDNA. Then, qRT-PCR was conducted following the protocol of the Real-time PCR Easy™-SYBR Green I Kit. At last, the software Bio-Rad CFX Maestro 1.0 was utilized to quantify iNOS and COX-2 expression, with GAPDH as the internal reference. Table S1 provides specific primer sequence information.

#### 2.7.5 Western blot assay

Following treatment of macrophages with the ‘Fengtang’ plum seed EE *via* the aforementioned method, nuclear and cytoplasmic proteins were separated using a cytoplasmic and nuclear protein extraction kit, and total proteins were isolated using RIPA lysis buffer. The BCA assay kit was then used to measure the protein concentration. Subsequently, 10% SDS-PAGE was used to separate 20 μg of protein, which was then transferred to a PVDF membrane. The membrane underwent three rounds of washing with TBS-T solution after being blocked for 1 h with 5% nonfat milk. Next, the membrane was treated with the primary antibody at 4 °C for the whole night. Following three TBS-T solution washes, the secondary antibody was utilized to blot the membrane for 1 h. After washing three times, protein bands were seen using ECL reagent, and Bio-Rad Image Lab software was utilized to measure the band intensities. All antibodies were diluted 1:1000 (*v*/v), with GAPDH as the internal reference gene.

#### 2.7.6 Immunofluorescence staining assay

Sterile slides were placed in the well center of 6-well plates. After injecting RAW264.7 macrophage suspension (1 mL, 6 × 10^5^ cells/mL) onto the slides and culturing for 24 h, three groups were established: control, LPS, and EE. Next, culture medium (1 mL) was pipetted into the control group and LPS group, and 1 mL of EE solution (250 μg/mL) was pipetted into the EE group. The supernatant was dumped out after the 2 h incubation period. The control group received 2 mL of culture medium, the LPS group received 2 mL of LPS (1 μg/mL), and the EE group received 2 mL of mixed solutions containing LPS (1 μg/mL) and EE (250 μg/mL). After incubation for 1 day and three washes with PBS, the slides were fixed for 15 min with 1 mL of 4% paraformaldehyde and closed using a blocking solution for 1 h. Subsequently, the slides were treated with the NF-κB p65 antibody overnight at 4 °C and the secondary antibody for 90 min in the dark. Thereafter, we stained nuclei with DAPI for 5 min after incubating slides with secondary antibodies labeled by Alexa Fluor 488 in the dark for 90 min. Macrophages were photographed by a Leica TCS SP8 laser confocal microscope (Leica Microsystems, Germany).

### 2.8 *In vivo* study of the anti-colitis effect of ‘Fengtang’ plum seed EE

#### 2.8.1 Experimental mice

SiPeiFu Biological Co., Ltd. (Beijing, China) supplied male C57BL/6 mice that were 6–8 weeks old and weighed 20 ± 2 g. The animal chamber was kept at SPF level (22–25 °C) with 12 h of light daily. During their acclimation period (7 days), mice were allowed free access to water and food. The Ethics Committee of Laboratory Animal Science of Guizhou University gave its approval to this experimental protocol (animal ethics No: EAE-GZU-2024-E042). All animal procedures complied with the Chinese National Laboratory Animal-Guideline for Ethical Review of Animal Welfare (GB/T 35892–2018) and the NIH (National Research Council) Guide for the Care and Use of Laboratory Animals.

#### 2.8.2 Mouse mode

Forty mice, ten in each of the four groups, were randomly assigned. Control group: mice had unrestricted access to distilled water and were administered normal saline (0.3 mL) *via* gastric gavage daily. DSS group: mice had free access to 4% DSS solution and were administered normal saline (0.3 mL) daily. Positive group: mice were granted unrestricted access to 4% DSS solution and gavaged mesalazine (0.3 mL, 200 mg/kg) daily. EE group: mice had free access to a 4% DSS solution and were administered daily with 0.3 mL (400 mg/kg) of EE solution. Every day, the stool consistency, blood in stool, and weight of mice were noted. After a 6 h fast and ether anaesthesia, they were murdered by cervical dislocation. Blood was collected from the eyeballs, and colon tissue was obtained by dissection. Eventually, photos were taken, and the colon's length was measured.

#### 2.8.3 DAI score

The DAI score is a standard scoring system comprising degrees of stool consistency, blood in stool, and weight loss. The scoring criteria are shown in Table S2.

#### 2.8.4 H&E staining of colon tissue

Colon tissue samples were preserved for 24 h using tissue fixative. Next, the tissue samples were embedded in paraffin after being dried. After sectioning the tissue into 4 μm thick sections, hematoxylin staining solution was used to stain the tissues. Distilled water was utilized to wash the slices. Then, slices were treated using hematoxylin differentiation solution, incubated in hematoxylin bluing solution, and washed again. Subsequently, 85% and 95% alcohol were utilized to dehydrate the slices. After that, the slices were stained using eosin staining solution, dewaxed with xylene, and sealed. Ultimately, imaging was performed using a Panoramic 250/MIDI scanner.

#### 2.8.5 Determination of IL-1β, MDA, IL-6, and TNF-α levels and CAT and SOD activities in serum and tissue fluid

For serum preparation, the serum was obtained by centrifuging blood samples for 20 min at 4 °C and 3000 g. For colonic tissue fluid preparation, 1 g of colon tissue in PBS (9 mL, pH 7.4) was homogenized, and centrifugation (3000 *g*) was performed at 4 °C for 20 min to collect the tissue fluid. IL-6, IL-1β, MDA, and TNF-α ELISA kits were used to determine their concentrations in tissue fluid and serum. CAT and SOD activity kits were utilized to determine the CAT and SOD activities, respectively.

### 2.9 Network pharmacology of ‘Fengtang’ plum seed EE

#### 2.9.1 Searching for potential targets for the treatment of colitis using bioactive compounds from ‘Fengtang’ plum seed EE

We screened the potential active compounds of ‘Fengtang’ plum seed EE using the SwissADME database (http://www.swissadme.ch/) and the Traditional Chinese Medicine Systems Pharmacology Database and Analysis Platform (TCMSP database, https://old.tcmsp-e.com/tcmsp.php). The DL value (drug-likeness), OB value (oral bioavailability), and gastrointestinal absorption were set to ≥0.18, ≥0.36, and high, respectively. In addition, the Simplified Molecular Input Line Entry System (SMILES) of potential active compounds was obtained by using the PubChem database (https://pubchem.ncbi.nlm.nih.gov/). We then imported the SMILES into SwissTargetPrediction (http://www.swisstargetprediction.ch/) and collected the potential targets of the active compounds. Subsequently, Therapeutic Target Database (TTD, https://db.idrblab.net/ttd/), PharmGKB database (https://www.pharmgkb.org/), OMIM database (https://www.omim.org/), and Genecards (https://www.genecards.org/) were utilized to search for potential target genes for colitis. Finally, overlap analysis of colitis-related targets and target genes of potential active compounds in ‘Fengtang’ plum seed EE was performed using Venny 2.1.0 (https://bioinfogp.cnb.csic.es/tools/venny/index.html) to obtain overlapping targets (Venny, 2007). These targets are all potential target genes for ‘Fengtang’ plum seed EE treatment of colitis.

#### 2.9.2 Construct protein-protein interaction (PPI) networks and drug-target networks

The STRING database was used to construct the PPI network of intersectional targets, with the species set to *Homo sapiens*, the minimum required interaction score set to high confidence (0.700), and disconnected nodes hidden. Subsequently, we further optimized and refined the PPI network *via* Cytoscape 3.9.1 software. Furthermore, Cytoscape 3.9.1 software was employed to construct a network of potential active compounds and targets for the treatment of colitis with ‘Fengtang’ plum seed EE. Next, the Centiscape 2.2 plug-in of the Cytoscape software was used to analyze the network, and core targets were selected as the targets for the next step of analysis based on the comprehensive ranking of node connectivity (degree), node closeness (closeness), and node betweenness (betweenness). Subsequently, Cytoscape software was employed to construct a network of potential active compounds and targets from ‘Fengtang’ plum seed EE to investigate its therapeutic mechanism for colitis. The nodes depicted ‘Fengtang’ plum seed EE, components, and target genes, with edges illustrating their relationships.

#### 2.9.3 Molecular docking

We validated the binding of the core target with the active compounds of ‘Fengtang’ plum seed EE through molecular docking. The PDB (RCSB Protein Data Bank, https://www.rcsb.org) provided the protein crystal structures. The TCMSP database included the original ligand files. We utilized Autodock 4.2 software to eliminate unnecessary water molecule hydrogenation and predict protein-ligand interactions, with the results visualized using PyMOL software.

### 2.10 Statistical analysis

The results are shown as means ± SD based on three separate assays. The statistical program IBM SPSS 25 was used to evaluate the data. To determine significant differences in TPC and TFC, a two-tailed unpaired *t*-test was used. ANOVA and LSD *post hoc* tests were used to assess significant differences in the other data, with *p < 0.05, **p < 0.01, ***p < 0.001.

## 3 Results

### 3.1 Compositional characterization of ‘Fengtang’ plum seed WE and EE

The yield of ‘Fengtang’ plum seed WE and EE separately were 1.42% and 0.95%. As shown in Fig. S1A, EE's TPC was 33.62 ± 0.04 mg GAE/g extract, whereas the TPC for WE was 38.91 ± 0.04 mg GAE/g extract. The TFC in WE and EE were 35.12 ± 0.06 and 61.72 ± 0.34 mg RE/g extract, correspondingly. As shown in Fig. S1B and C, the DPPH and ABTS tests were utilized to evaluate the free radical scavenging capabilities of ‘Fengtang’ plum seed WE and EE. The IC_50_ values of WE were 109.99 ± 8.22 μg/mL for ABTS and 66.03 ± 2.89 μg/mL for DPPH. The IC_50_ values of EE were 132.63 ± 5.88 μg/mL for ABTS and 87.40 ± 1.13 μg/mL for DPPH. Both extracts showed mild free radical scavenging activity.

As shown in Table 1 and Fig. S2, fifty-one compounds were identified in ‘Fengtang’ plum seed extracts using UHPLC-Q-Orbitrap MS, including 18 phenolic compounds and 8 flavonoids (detailed analysis of fragmentation patterns in Supplementary Material). As illustrated in Fig. S3, the 18 identified phenolic compounds were 2-hydroxy-4-methoxybenzaldehyde (1), 6-hydroxyindole (9), protocatechuic acid (14), gentisic acid (15), vanillin (16), ethyl ferulate (17), orsellinic acid (18), 3,5-dimethoxy-4-hydroxybenzaldehyde (26), methyl vanillate (27), scopoletin (30), ferulaldehyde (33), sinapyl aldehyde (35), isofraxidin (36), ethyl 3,4-dihydroxybenzoate (37), aurantio-obtusin (41), fraxetin (42), *p*-hydroxybenzaldehyde (44), and 6-gingerol (46). The 8 flavonoids included prim-*O*-glucosylcimifugin (24), retrochalcone (25), kaempferol-3-*O*-rutinoside (28), medicarpin (31), procyanidin A2 (32), quercitrin (38), engeletin (40), and hesperetin (45).

**Table 1:** Phytochemical compounds detected in ‘Fengtang’ plum seed WE and EE using UHPLC-Q-Orbitrap-MS.

Peak	RT (min)	Identification^a^	Formula	[M + H]^+^(*m*/*z*)	[M-H]^−^(m/z)	Error ppm	MS^2^ fragment ions	WE^b^	EE^b^
1	0.168	2-Hydroxy-4-methoxybenzaldehyde	C_8_H_8_O_3_	153.05426		−2.4	135.07983, 125.10722, 111.09168, 109.06481, 93.07028	–	√
2	0.793	Quinic acid	C_7_H_12_O_6_		191.05464	–3.1	173.04425, 127.03848, 111.00716, 109.02780, 93.03295	√	√
3	0.794	D-Gluconic acid	C_6_H_12_O_7_		195.04959	−2.6	177.03900, 129.0177, 99.00717, 87.00715, 75.00718	√	√
4	0.891	Sucrose	C_12_H_22_O_11_		341.10748	−4.3	179.05460, 161.04405, 113.02273, 101.02281, 89.02279	–	√
5	1.041	Mannitol	C_6_H_14_O_6_		181.07027	−2.8	163.05959, 119.03339, 101.02279, 89.02278, 71.01226	√	√
6	1.279	Citric acid	C_6_H_8_O_7_		191.01834	−3.1	173.04670, 129.01770, 111.00712, 87.00711, 85.02786	√	√
7	1.901	Scopolin	C_16_H_18_O_9_	355.0988		−9.6	325.08966, 324.07996, 193.04662, 192.09862, 177.01590	√	–
8	2.092	L-Phenylalanine	C_9_H_11_NO_2_	166.08595		−1.8	149.05951, 131.04883, 120.08081, 103.05437, 91.05443	√	√
9	2.184	6-Hydroxyindole	C_8_H_7_NO	134.05984		−1.5	107.04916, 106.06535, 91.05457, 79.05469, 77.03918	√	√
10	2.462	5-Hydroxymethylfurfural	C_6_H_6_O_3_	127.03885		−0.9	109.02841, 99.04416, 97.02861, 81.03387, 67.93543	√	–
11	2.766	L-Tryptophan	C_11_H_12_N_2_O_2_	205.09659		−2.8	188.06993, 170.05952, 146.05956, 144.08025, 118.06496	√	–
12	2.888	Clareolide	C_16_H_26_O_2_	251.19995		−2.4	203.06937, 191.03285, 161.05884, 107.04897, 98.98437	√	–
13	2.913	3-Furfuryl 2-pyrrolecarboxylate	C_10_H_9_NO_3_	192.06494		−3.0	174.05452, 164.06984, 148.07512, 147.06732, 146.05946	√	–
14	2.983	Protocatechuic acid	C_7_H_6_O_4_		153.01782	−5.2	153.01776, 109.02789, 128.02004, 91.01730	–	√
15	3.504	Gentisic acid	C_7_H_6_O_4_		153.01781	−9.9	123.04301, 109.02789, 108.02004, 91.01730, 81.03304	√	√
16	4.354	Vanillin	C_8_H_8_O_3_		151.03850	−5.3	125.05949, 111.04411, 110.03619, 93.03375, 65.03922	√	√
17	4.548	Ethyl ferulate	C_12_H_14_O_4_	223.09596		−2.4	165.05411, 147.04362, 123.04387, 119.04903, 91.05450	√	–
18	5.882	Orsellinic acid	C_8_H_8_O_4_	169.04907		−2.8	154.02557, 141.05417, 126.03088, 111.04404, 109.02842	√	–
19	5.966	*o*-Veratraldehyde	C_9_H_10_O_3_	167.06992		−2.1	137.05923, 124.05167, 123.04388, 106.04147, 78.04688	√	–
20	6.45	2-Isopropylmalic acid	C_7_H_12_O_5_		175.05969	−3.4	157.04906, 131.06981, 115.03846, 113.05918, 85.06428	√	√
21	6.769	Anisic aldehyde	C_8_H_8_O_2_	137.05934		−2.7	122.0361, 107.0491, 105.03357, 94.04154, 91.05454	√	–
22	8.67	Leucinic acid	C_6_H_12_O_3_		131.06972	−5.9	113.05937, 87.04353, 85.06423, 69.03291	√	√
23	8.933	Aucubin	C_15_H_22_O_9_		391.12305 [M + HCOO]-	−3.9	168.04134, 161.04401, 153.01781, 150.02994, 71.01222	√	√
24	9.933	Prim-*O*-glucosylcimifugin	C_22_H_28_O_11_	469.16864		−3.8	289.06860, 275.08969, 259.07739, 232.06985, 185.04170	√	–
25	10.987	Retrochalcone	C_16_H_14_O_4_	271.09555		−3.5	211.07454, 193.06244, 183.07977, 165.06931, 153.06932	√	–
26	11.247	3,5-Dimethoxy-4-hydroxybenzaldehyde	C_9_H_10_O_4_	183.06538		1.1	155.06973, 140.04636, 123.04394, 95.04944	√	√
27	11.268	Methyl vanillate	C_9_H_10_O_4_		181.04916	−3.5	163.05963, 151.00209, 123.00710, 101.02277, 89.02277	√	√
28	11.338	Kaempferol-3-*O*-rutinoside	C_27_H_30_O_15_	595.16156		−6.2	331.07742, 287.07294, 167.01352, 147.04373, 138.14183	√	–
29	11.388	4-Methoxyphenylacetic acid	C_9_H_10_O_3_		165.05414	−4.9	147.04352, 135.04373, 121.02785, 119.04856, 93.03297	–	√
30	11.862	Scopoletin	C_10_H_8_O_4_	193.04907		−2.4	161.05922, 137.05928, 133.06447, 115.05415, 105.06998	–	√
31	12.102	Medicarpin	C_16_H_14_O_4_	271.09558		−3.3	221.07463, 183.07990, 165.09637, 153.06934, 119.04904	√	–
32	12.229	Procyanidin A2	C_30_H_24_O_12_		575.11755	−3.4	449.08585, 289.07065, 285.03922, 125.02278, 109.02790	√	√
33	12.884	Ferulaldehyde	C_10_H_10_O_3_	179.06973		−3.0	161.05917, 147.04358, 119.04901, 105.06992, 91.05450	√	√
34	13.354	Sauchinone	C_20_H_20_O_6_	357.13199		−3.6	339.12033, 327.12204, 219.07930, 203.06931, 137.05922	√	–
35	13.409	Sinapyl aldehyde	C_11_H_12_O_4_	209.08044		−1.9	191.06963, 181.08524, 177.05406, 149.05931, 121.06463	√	√
36	13.792	Isofraxidin	C_11_H_10_O_5_		221.04422	−2.2	206.02063, 177.05414, 162.03069, 134.03569, 106.04064	√	√
37	13.873	Ethyl 3,4-dihydroxybenzoate	C_9_H_10_O_4_		181.04913	−2.5	153.01770, 152.00981, 124.01492, 109.02784, 89.02277	–	√
38	13.924	Quercitrin	C_21_H_20_O_11_		447.09140	−4.2	300.02628, 271.02368, 255.02866, 243.02863, 151.00201	–	√
39	13.947	Azelaic acid	C_9_H_16_O_4_		187.09607	−2.7	169.08546, 143.10611, 125.09556, 97.06422, 57.03305	√	√
40	14.486	Engeletin	C_21_H_22_O_10_		433.11209	−4.5	167.03334, 152.00984, 123.04343, 121.02779, 108.01998	√	–
41	15.153	Aurantio-obtusin	C_17_H_14_O_7_	331.08026		−2.9	313.10608, 285.11072, 253.84850, 225.09013, 210.06685	√	–
42	15.269	Fraxetin	C_10_H_8_O_5_	209.04390		−2.6	194.09346, 191.06967, 177.05403, 149.05930, 121.06462	–	√
43	15.316	*β*-Asarone	C_12_H_16_O_3_	209.11670		−2.5	181.08531, 177.05402, 149.05930, 131.04886, 106.04140	√	√
44	15.968	*p*-Hydroxybenzaldehyde	C_7_H_6_O_2_		121.02782	−8.1	121.02782, 108.02002, 93.03271	√	–
45	16.057	Hesperetin	C_16_H_14_O_6_		301.07077	−3.3	286.04721, 285.03928, 269.04456, 258.05203, 243.02878	√	√
46	22.093	6-Gingerol	C_17_H_26_O_4_		293.17462	−4.1	236.10406, 221.15331, 205.12192, 192.11400, 177.09044	√	–
47	22.483	Evodiamine	C_19_H_17_N_3_ O	304.14349		−3.1	171.09106, 144.08037, 134.05972, 106.06517, 91.05452	–	√
48	23.095	Senkyunolide A	C_12_H_16_O_2_	193.12180		−2.6	161.05911, 137.05930, 133.06448, 105.06992, 79.05479	–	√
49	26.349	Methyl hexadecanoate	C_17_H_34_O_2_		315.25275 [M + HCOO]-	−0.7	313.23737, 297.24237, 171.10121, 141.12677, 127.11147	–	√
50	27.612	Germacrone	C_15_H_22_O	219.17380		−2.5	201.16324, 177.12686, 163.11133, 149.09575, 135.08006	–	√
51	28.089	*α*-Linolenic acid	C_18_H_30_O_2_	279.23117		−2.5	123.11663, 95.08578, 81.07032, 79.05468, 67.05484	–	√

^a^Phytochemical identification was performed based on comparison with references and the mzVault and mzCloud databases. Detailed analyses of MS^2^ fragments are shown in the Supplementary Material. ^b^ “-” indicates that the phytochemical component was not detected in ‘Fengtang’ plum seed extracts. “√” indicates that the phytochemical component was detected in ‘Fengtang’ plum seed extracts.

### 3.2 Anti-inflammatory action *in vitro*

#### 3.2.1 Cytotoxicity and inflammatory factor regulation of ‘Fengtang’ plum seed extracts

**Fig. 1:**
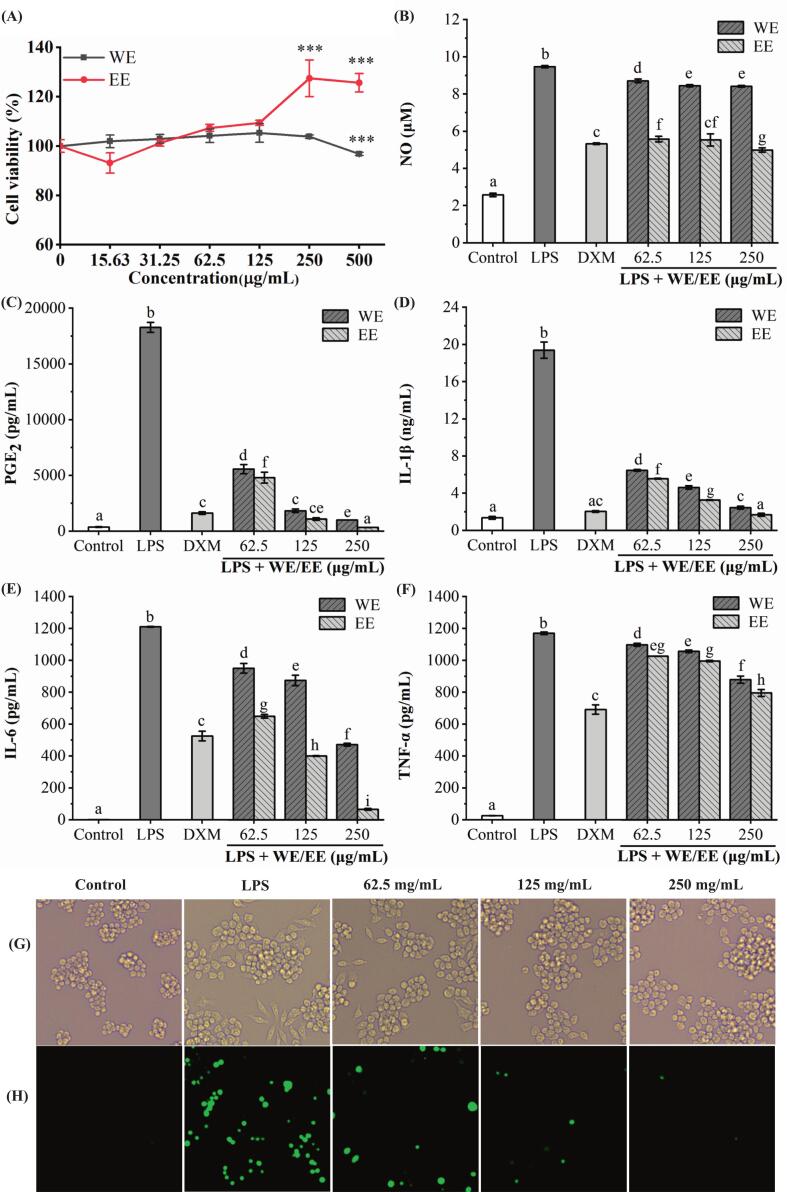
The impact of ‘Fengtang’ plum seed WE and EE on inflammatory factor release in RAW264.7 macrophage with LPS stimulation and the influence of ‘Fengtang’ plum seed EE on LPS-stimulated cell morphology and ROS level. (A) WE and EE's cytotoxic effects on RAW264.7 macrophages. (B) NO kit tested the release of NO. (C—F) ELISA kits detected PGE_2_ (C), IL-1β (D), IL-6 (E), and TNF-α (F) contents in cell supernatants. (G) The morphology of macrophages was observed using an inverted microscope. (H) After staining with the DCFH-DA fluorescent probe, intracellular ROS was examined using fluorescence microscopy. In the cytotoxicity assay, the asterisk is used to indicate significant differences *versus* untreated cells (***p < 0.001). In the detection of inflammatory factor release, significant differences are denoted by different letters (*p* < 0.05).

‘Fengtang’ plum seed WE and EE's cytotoxicity on RAW264.7 cells was evaluated using the MTT test (Fig. 1A). WE and EE (15.63–250 μg/mL) exhibited no evident cytotoxicity toward RAW264.7 cells compared with the untreated group. Therefore, for subsequent experiments, doses of 250, 125, and 62.5 μg/mL were used. As illustrated in Fig. 1B-F, WE and EE groups significantly reduced NO, PGE_2_, IL-6, IL-1β, and TNF-α contents in cell supernatants in comparison to the LPS group. In addition, at the same dosage, EE had a stronger inhibitory impact on the production of these inflammatory factors than WE (*p* < 0.05). These findings indicated that EE had a greater anti-inflammatory impact than WE. Hence, subsequent experiments were conducted to further investigate ‘Fengtang’ plum seed EE's anti-inflammatory action.

#### 3.2.2 Impact of ‘Fengtang’ plum seed EE on ROS generation

As illustrated in Fig. 1G, cells in the control group were regular spherical. However, cells treated with LPS showed obvious morphological changes and became spindle-shaped. EE pretreatment reduced the number of spindle cells. As illustrated in Fig. 1H, LPS treatment led to a considerable increase in the number of green fluorescent RAW264.7 macrophages as compared to the control group. However, EE pretreatment gradually decreased the amount of green fluorescent cells, indicating that it suppressed ROS generation.

#### 3.2.3 ‘Fengtang’ plum seed EE inhibited LPS-stimulated iNOS and COX-2 expression and inflammatory cytokine mRNA expression

**Fig. 2:**
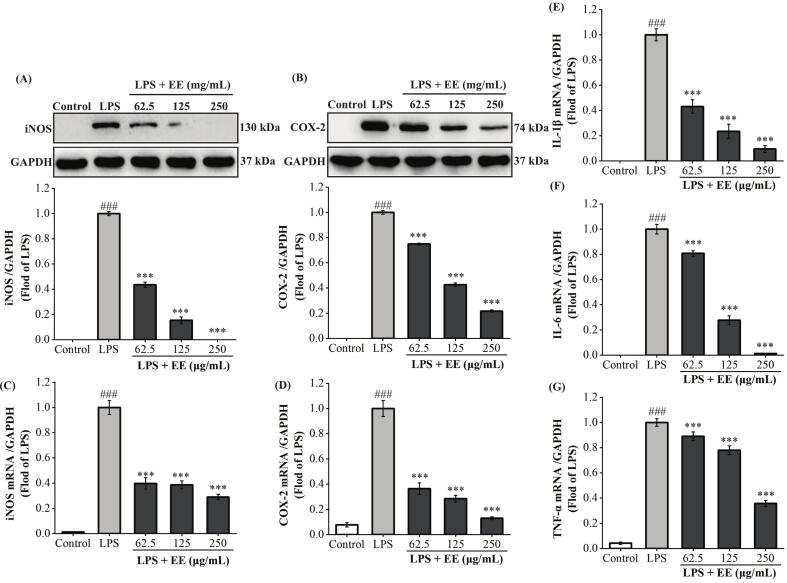
Effects of ‘Fengtang’ plum seed EE on LPS-stimulated iNOS and COX-2 expression as well as inflammatory cytokine mRNA expression. (A, B) Western blot detection of the iNOS (A) and COX-2 (B) protein levels. (C-G) qRT-PCR determination of iNOS (C), COX-2 (D), IL-1β (E), IL-6 (F), and TNF-α (G) mRNA expression levels. GAPDH served as the internal control. In comparison to the control group, ###*p* < 0.001. ***p < 0.001 *vs.* LPS group.

As exhibited in Fig. 2A-D, the COX-2 and iNOS protein and mRNA expressions were examined utilizing the western blot and qRT-PCR. Their transcription and translation levels in the LPS group were substantially higher than those in the control group (*p* < 0.001). In a dose-dependent manner, EE considerably decreased the COX-2 and iNOS protein and mRNA expression levels in comparison to the LPS group. Hence, ‘Fengtang’ plum seed EE repressed LPS-induced PGE_2_ and NO oversecretion by inhibiting COX-2 and iNOS transcription and translation levels. As exhibited in Fig. 2E-G, the LPS group exhibited significantly higher inflammatory cytokine mRNA expression levels (IL-1β, IL-6, and TNF-α) than the control group (*p* < 0.001). However, EE considerably inhibited their mRNA expression induced by LPS. According to the aforementioned findings, ‘Fengtang’ plum seed EE repressed IL-1β, IL-6, and TNF-α overproduction by blocking their transcription.

#### 3.2.4 Impact of ‘Fengtang’ plum seed EE on NF-κB and MAPK activation

**Fig. 3:**
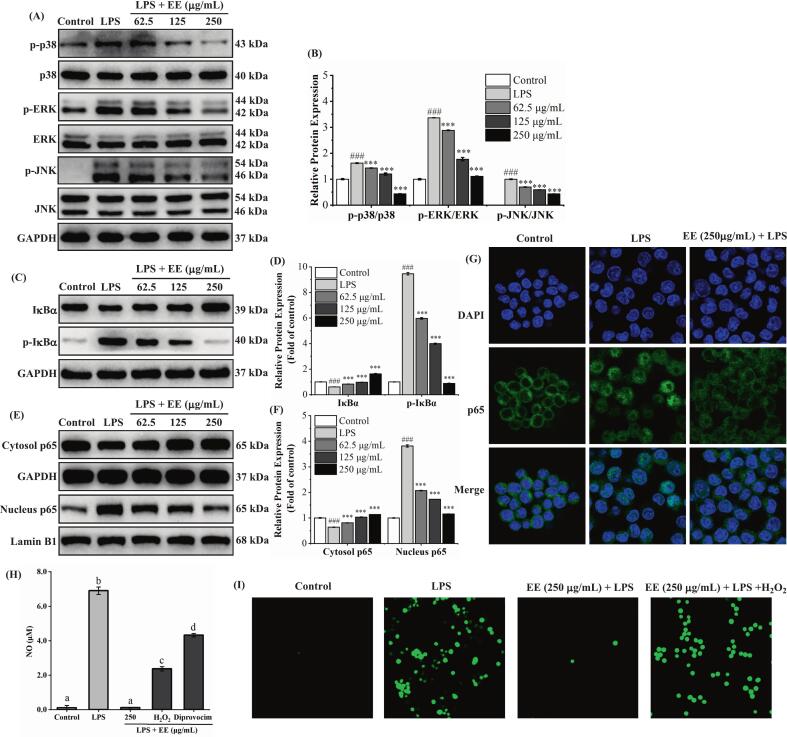
Effects of ‘Fengtang’ plum seed EE on activation of the MAPK/NF-κB pathway triggered by LPS. (A, B) The phosphorylated and total p38, ERK, and JNK proteins were measured using a western blot. (C, D) Western blot analysis of the amounts of IκBα and p-IκBα proteins. (E, F) Western blot detection of the protein amounts of cytosol p65 and nucleus p65. The internal reference was GAPDH. (G) Immunofluorescence staining was utilized to determine the impact of ‘Fengtang’ plum seed EE on LPS-induced NF-κB nuclear translocation. (H) NO levels after using H_2_O_2_ (ROS activator) and diprovocim (MAPK and NF-κB pathway activator). (I) ROS levels after using H_2_O_2_ (ROS activator). In contrast with the control group, ###*p* < 0.001. **p < 0.01, ***p < 0.001 in comparison to the LPS group.

The ERK, p-ERK, JNK, p-JNK, p38, and p-p38 protein expression levels were assessed by Western blot. As depicted in Fig. 3A and B, pretreatment with ‘Fengtang’ plum seed EE significantly reduced the phosphorylation levels of JNK, p38, and ERK (*p* < 0.001). Additionally, ‘Fengtang’ plum seed EE considerably reduced the degradation of IκBα and down-regulated the phosphorylation of IκBα (*p* < 0.001) (Fig. 3C and D). As exhibited in Fig. 3E and F, the EE groups had considerably higher levels of p65 in the cytoplasm than the LPS group, whereas their nucleus had significantly lower levels of p65 (*p* < 0.001). As depicted in Fig. 3G, the control group's p65 (green fluorescence) was found surrounding the cell nucleus (blue fluorescence), but the LPS group's p65 was mostly located in the nucleus. However, the EE (250 μg/mL) group's p65 was mostly localized in the cytoplasm. Our findings demonstrated that ‘Fengtang’ plum seed EE suppressed the activation of the MAPK (through preventing JNK, ERK, and p38 phosphorylation) and NF-κB (through blocking the p65 nuclear translocation and IκBα degradation and phosphorylation) pathways.

In addition, H_2_O_2_ (ROS activator) and diprovocim (MAPK and NF-κB pathway activator) were used to confirm whether EE exerts its anti-inflammatory effect by regulating the ROS/MAPK/NF-κB pathway. As shown in Fig. 3H, compared with the EE group (250 μg/mL), treatment with H_2_O_2_ or diprovocim remarkably reversed the inhibitory effect of EE on LPS-induced NO production. Furthermore, as depicted in Fig. 3I, compared with the EE group, the number of green fluorescent cells significantly increased after H_2_O_2_ treatment, indicating that H_2_O_2_ reversed the ROS-scavenging effect of EE. These results further confirmed that EE exerted its anti-inflammatory effect through the ROS/MAPK/NF-κB pathway.

### 3.3 Effects of ‘Fengtang’ plum seed EE on DSS-induced colitis

#### 3.3.1 ‘Fengtang’ plum seed EE alleviated DSS-induced colitis

**Fig. 4:**
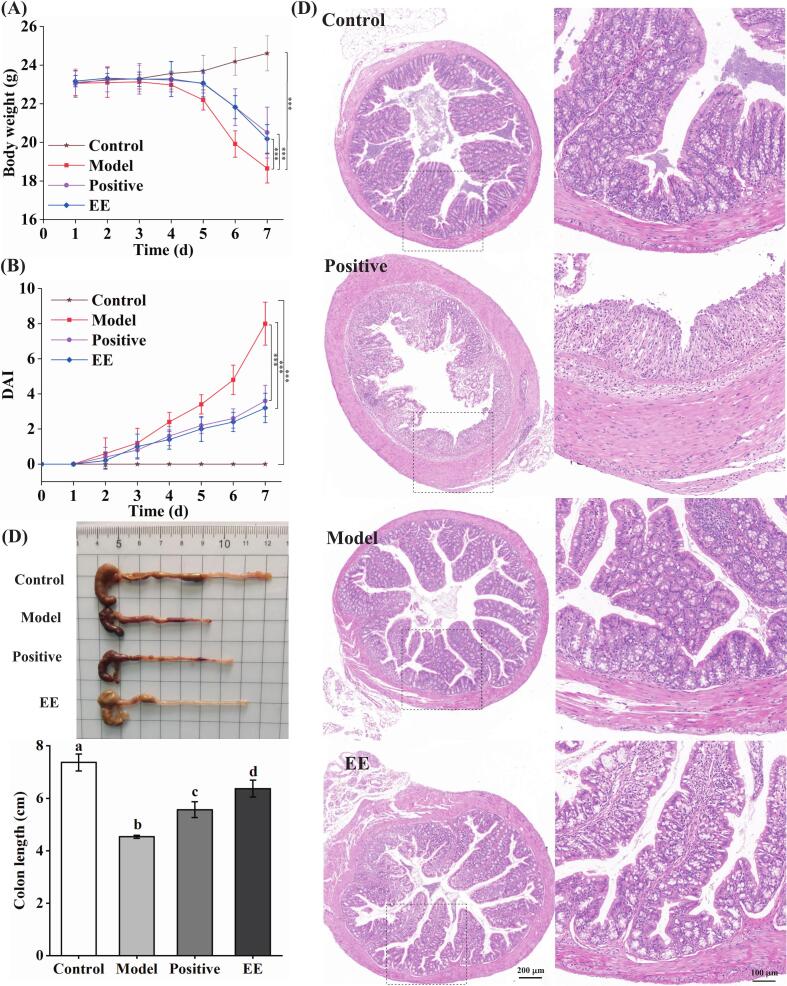
Effect of ‘Fengtang’ plum seed EE on DSS-induced colitis. (A-C) Effects of ‘Fengtang’ plum seed EE on body weight (A), DAI scores (B), and colon length (C). (D) H&E-stained images of mouse colon tissue. In the body weight and DAI assay, significant differences were indicated with the asterisk (***p < 0.001). Furthermore, in the colon length, distinct letters signify a significant difference (*p* < 0.05).

As presented in Fig. 4A and B, the body weight of the model group markedly decreased, and DAI distinctly increased on the seventh day in comparison to the control group. The weight loss of mice given EE and mesalazine was significantly relieved, and their DAI scores were obviously decreased in comparison to the model group. As presented in Fig. 4C, EE significantly inhibited the shortening of colon length (*p* < 0.05). Furthermore, H&E staining observed colon tissue injury in mice (Fig. 4D). The control group's colon sections exhibited a normal structure. However, the DSS group showed severe damage to colonic tissue, including crypt destruction, a reduction in goblet cells, and considerable infiltration of inflammatory cells. ‘Fengtang’ plum seed EE (250 μg/mL) relieved DSS-induced colonic injury. These findings suggested that ‘Fengtang’ plum seed EE effectively alleviated DSS-induced colonic tissue damage.

#### 3.3.2 ‘Fengtang’ plum seed EE suppressed oxidative stress indices and inflammatory cytokine levels in tissues and serum

**Fig. 5:**
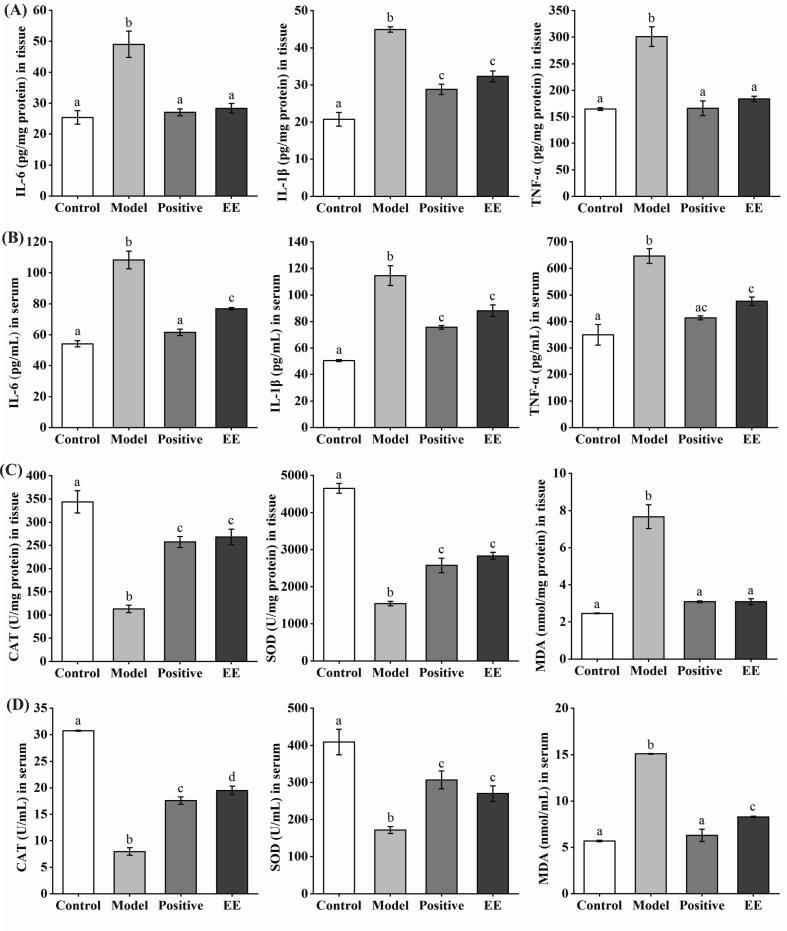
Effects of ‘Fengtang’ plum seed EE on the inflammatory cytokine levels and oxidative stress indices in tissue fluid and serum of mice induced by DSS. (A, B) Effects of ‘Fengtang’ plum seed EE on IL-1β, IL-6, and TNF-α concentrations in mouse tissue fluid and serum. (C, D) Effects of ‘Fengtang’ plum seed EE on the MDA content and enzymatic activities of CAT and SOD in mouse tissue fluid and serum. Substantial variances are indicated by distinct letters on the bars (*p* < 0.05).

As displayed in Fig. 5A and B, in comparison to the control group, DSS treatment markedly elevated these inflammatory cytokine levels (IL-1β, IL-6, and TNF-α) in mouse tissue fluid and serum. ‘Fengtang’ plum seed EE effectively decreased inflammatory cytokine levels. As displayed in Fig. 5C and D, EE effectively inhibited ROS production by raising CAT and SOD activities and decreasing MDA concentration as compared to the model group. These findings showed that ‘Fengtang’ plum seed EE alleviated DSS-induced colitis in mice by decreasing inflammatory cytokine levels and ROS generation.

### 3.4 Network pharmacology analysis

#### 3.4.1 Determination of core targets of ‘Fengtang’ plum seed EE to treat colitis

**Fig. 6:**
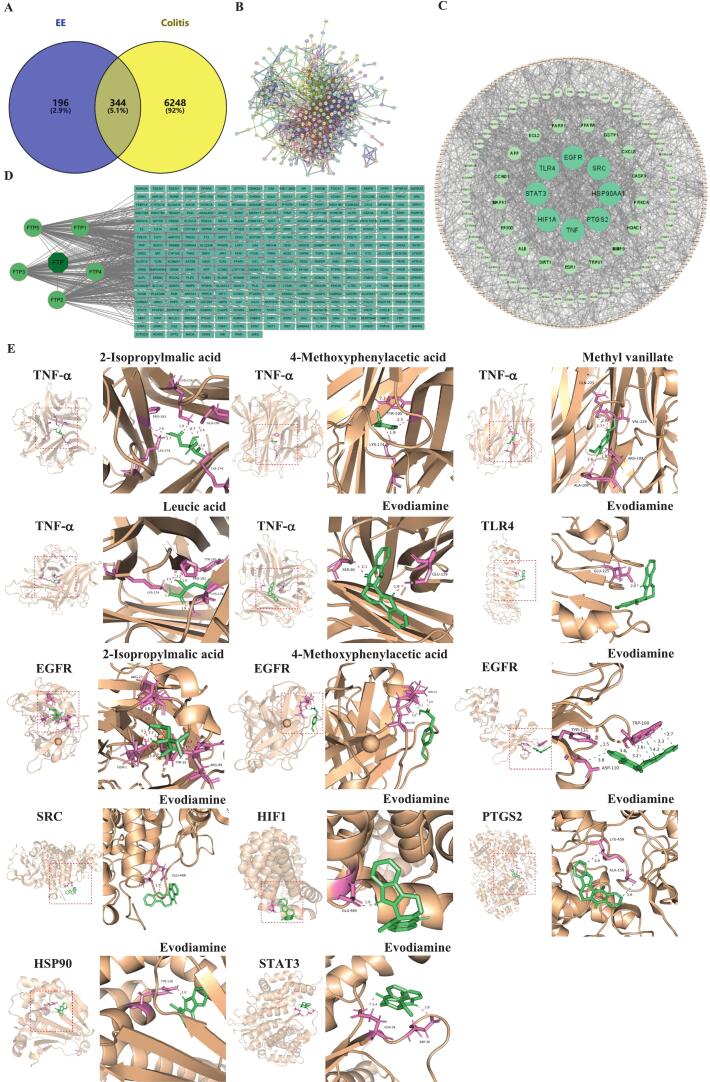
Network pharmacology analysis of ‘Fengtang’ plum seed EE in treating colitis and the binding modes of potential active compounds with core targets. (A) The Venn diagram of ‘Fengtang’ plum seed EE's colitis-associated genes and target genes. (B) The PPI network diagram. (C) The PPI network diagram is displayed in Cytoscape 3.9.1. (D) Network diagram of ‘Fengtang’ plum seed EE-potential active compounds-target network. ‘Fengtang’ plum seed EE was expressed as FTP. The potential active components in ‘Fengtang’ plum seed EE were as follows: 2-isopropylmalic acid (FTP1), leucinic acid (FTP2), methyl vanillate (FTP3), 4-methoxyphenylacetic acid (FTP4), and evodiamine (FTP5). (E) Binding modes of potential active compounds with core targets.

Five potential active compounds (2-isopropylmalic acid, leucinic acid, methyl vanillate, 4-methoxyphenylacetic acid, and evodiamine) were screened out from the database. In total, 540 targets were obtained from the SwissTargetPrediction and TCMSP databases. From the aforementioned four disease databases, 6592 genes were identified as potential targets for colitis. Based on a Venn diagram, 344 overlapping targets were identified as potential therapeutic targets in Fig. 6A. The STRING was utilized to build a PPI network consisting of 344 overlapping target genes (Fig. 6B). ‘Fengtang’ plum seed EE-potential active components-targets links were revealed by constructing and visualizing the network using Cytoscape 3.9.1. Subsequently, the Cytoscape revealed clearer relationships and identified core targets (Fig. 6C). As shown in Fig. 6D, the network was composed of 332 nodes and 505 edges. In the network, the core targets were identified as TNF-α, EGFR, SRC, HIF1, COX-2, HSP90, STAT3, and TLR4.

#### 3.4.2 Molecular docking results

Based on network pharmacology analysis results, molecular docking was performed using AutoDock 4.2 to confirm the interactions between bioactive compounds and their core targets. A binding affinity below −5 kcal/mol indicates strong binding of the compound to the target (Ran et al., 2024). As shown in Fig. 6E and Table S3, the binding energies of TNF-α to 2-isopropylmalic acid, leucinic acid, methyl vanillate, 4-methoxyphenylacetic acid, and evodiamine were − 5.44, −5.74, −5.8, −5.81, and − 7.44 kcal/mol, respectively. The binding energies of EGFR to 2-isopropylmalic acid, 4-methoxyphenylacetic acid, and evodiamine are −5.98, −6.21, and − 6.92 kcal/mol, respectively. The binding energies of evodamine to SRC, HIF1, PTGS2 (COX-2), HSP90, STAT3, and TLR4 were − 5.14, −6, −5.63, −7.87, −5.87, and − 7.46 kcal/mol, respectively.

## 4 Discussion

Our study showed that ‘Fengtang’ plum seed was abundant in phenolic and flavonoid compounds. Additionally, phytochemical analysis using UHPLC-Q-Orbitrap-MS revealed 51 compounds, containing 18 phenolic compounds and 8 flavonoid compounds. Previous studies on the chemical composition of plum seed from other cultivated varieties have confirmed that it was abundant in phenolic and flavonoid compounds (Khallouki et al., 2012; Savić et al., 2016). Compared with previous studies on the chemical composition of plum seed, except for vanillin, the other 50 compounds in this study were identified from ‘Fengtang’ plum seed for the first time. Our research and the above studies have shown that ‘Fengtang’ plum seed is abundant in phenolic and flavonoid compounds.

Prior studies have demonstrated that the scavenging effect on ABTS and DPPH free radicals can preliminarily indicate that it has the ability to scavenge ROS *in vivo* and *in vitro* (Gupta, 2015). In the DPPH and ABTS experiments, our results showed that ‘Fengtang’ plum seed EE had free radical scavenging effects. At the same time, in LPS-induced RAW264.7 cells, ‘Fengtang’ plum seed EE dramatically inhibited the generation of ROS. In mice with DSS-stimulated colitis, ‘Fengtang’ plum seed EE reduced ROS levels by decreasing the MDA content as well as enhancing the activities of SOD and CAT in tissue fluid and serum. Therefore, our study showed that ‘Fengtang’ plum seed EE has the ability to scavenge ROS both *in vivo* and *in vitro*.

Inflammatory cytokine overproduction triggers a hyper-inflammatory response (Elenkov et al., 2005). Thus, we measured the levels of IL-1β, TNF-α, and IL-6 in RAW264.7 macrophages activated by LPS. Our results demonstrated that ‘Fengtang’ plum seed EE inhibited the IL-6, IL-1β, and TNF-α synthesis by downregulating their gene expression. iNOS and COX-2 are key rate-limiting enzymes for the generation of NO and PGE_2_, and inhibiting the activity of those enzymes can achieve the effect of treating colitis (Moita et al., 2013). In this study, ‘Fengtang’ plum seed EE repressed the secretion of inflammatory mediators (PGE_2_ and NO) by down-regulating the expression of COX-2 and iNOS genes and proteins. The MAPK/NF-κB pathway is essential in modulating inflammatory factor formation (Long et al., 2026). According to our research, ‘Fengtang’ plum seed EE inhibited ERK, JNK, and p38 phosphorylation, which in turn suppressed MAPK pathway activation. Additionally, it suppressed phosphorylation and degradation of IκBα, preventing NF-κB nuclear translocation and thereby curbing NF-κB activation. Previous research has shown that accumulating ROS could activate the MAPK/NF-κB pathway (Wan et al., 2022). Hence, our results indicated that ‘Fengtang’ plum seed EE inhibited the ROS/MAPK/NF-κB pathway, leading to a decrease in the inflammatory factor production. In addition, previous studies have confirmed that H_2_O_2_ can stimulate cells to produce ROS, while diprovocim can activate the MAPK and NF-κB signaling pathways (Wang et al., 2016; Xu et al., 2025). Our rescue experiments demonstrated that both H_2_O_2_ and diprovocim significantly reversed the inhibitory effect of EE on NO production. At the same time, H_2_O_2_ also reversed the EE's ROS-scavenging effect. These findings further demonstrated that the anti-inflammatory effects of EE were mediated by the inhibition of the ROS/MAPK/NF-κB pathway.

**Fig. 7:**
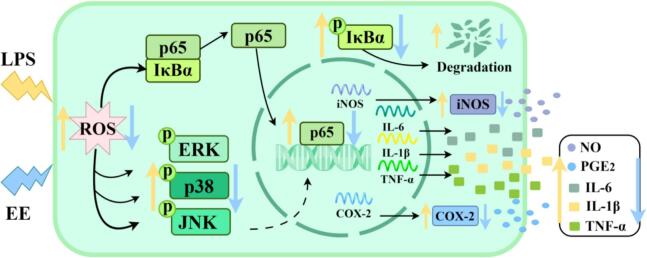
The anti-inflammatory mechanism of ‘Fengtang’ plum seed EE by inhibiting ROS/MAPK/NF-κB pathway.

As shown in Fig. 7, ‘Fengtang’ plum seed EE inhibited LPS-stimulated ROS generation, which in turn curbed the activation of NF-κB (by inhibiting IκBα phosphorylation and degradation, as well as blocking p65 nuclear translocation) and MAPK (by blocking p38, ERK, and JNK phosphorylation) pathways. Furthermore, it reduced the secretion of inflammatory mediators (PGE_2_ and NO) by suppressing the expression of COX-2 and iNOS genes and proteins. Additionally, it lowered inflammatory cytokine levels (IL-6, IL-1β, and TNF-α) by decreasing their transcription levels.

In DSS-induced colitis, ‘Fengtang’ plum seed EE significantly alleviated the symptoms of mice, relieved weight loss, inhibited the increase of DAI value, and ameliorated the shortening of colon length. The pathophysiology of colitis is tightly linked to oxidative stress indices (CAT, SOD, and MDA) and inflammatory cytokines (IL-6, IL-1β, and TNF-α) (da Costa Gonçalves et al., 2017; Guimbaud et al., 1998). Our study showed that ‘Fengtang’ plum seed EE lowered the generation of ROS (by strengthening CAT and SOD activities and decreasing MDA content), and decreased IL-6, IL-1β, and TNF-α contents in serum and colonic tissue fluid. These results were consistent with the above-mentioned *in vitro* anti-inflammatory experiment results, indicating that ‘Fengtang’ plum seed EE alleviated DSS-induced colonic injury by reducing ROS generation and inflammatory factor levels in colon tissue fluid and serum.

Network pharmacology was further employed to investigate the anti-colitis mechanism of the chemical substances identified in the ‘Fengtang’ plum seed EE. Five potential active compounds (2-isopropylmalic acid, leucinic acid, methyl vanillate, 4-methoxyphenylacetic acid, and evodiamine) and eight core targets (TNF-α, EGFR, SRC, HIF1, COX-2, HSP90, STAT3, and TLR4) were screened out. TNF-α, an inflammatory cytokine, binds to the receptor TNFR1 on the cell membrane, activates the MAPK/NF-κB pathway, and ultimately promotes the synthesis of various inflammatory cytokines; besides, TNF-α inhibitors are widely used to treat colitis (Bank et al., 2019; Zheng et al., 2024). In DSS-induced colitis, EGFR is activated and promotes the development of inflammation by modulating NF-κB pathway (Zhao et al., 2024). As a tyrosine kinase, Src is an upstream regulator of MAPK/NF-κB pathway activation (Xue et al., 2023). STAT3 promotes NF-κB activation by acetylating RelA and has been a key therapeutic target in colitis (Sugimoto, 2008; Lee et al., 2009a). HIF-1α can trigger NF-κB-dependent inflammation and participates in the pathogenesis of colitis (Qian et al., 2019; Walmsley et al., 2005). PTGS2 (COX-2) is an enzyme crucial for synthesizing PGE_2_, and COX-2 inhibitors have been shown to improve colitis in animal models (Li et al., 2018; Martín et al., 2005). HSP90 binds to Act1, mediating the transmission of IL-17 signaling, thereby activating the NF-κB pathway (Sønder et al., 2011; Wu et al., 2025). LPS binds to the transmembrane receptor TLR4, triggering an inflammatory response in macrophages by activating the NF-κB and MAPK pathways (Ciesielska et al., 2021). Besides, suppression of TLR4 has been considered as an effective target to treat colitis (Dai et al., 2022). Accordingly, ‘Fengtang’ plum seed EE may modulate the development of colitis by regulating these eight targets (TNF, EGFR, SRC, HIF1, PTGS2, HSP90, STAT3, and TLR4).

Previous research indicated that 2-isopropylmalic acid prevented the overproduction of inflammatory cytokines (TNF-α and IL-6) and ROS (Bae et al., 2021). Leucinic acid alleviated inflammation by preventing the release of inflammatory cytokines (Lee et al., 2024). Methyl vanillate has antioxidant properties and reduces the expression of inflammation-related cytokines (TNF-α and IL-6) (Lim et al., 2016). Evodiamine relieved colitis by controlling the release of inflammatory cytokines by inhibiting the NF-κB pathway (Zhang et al., 2022). Thus, these active chemicals may be responsible for the capacity of ‘Fengtang’ plum seed EE to relieve colitis.

Moreover, several phenolic and flavonoid compounds identified from ‘Fengtang’ plum seed EE have been shown to exert anti-inflammatory effects and/or alleviate colitis by inhibiting the activation of the ROS/MAPK/NF-κB pathway. For example, 6-gingerol, protocatechuic acid, fraxetin, ferulaldehyde, quercetin, procyanidin A2, and isofraxidin achieved anti-inflammatory effects by inhibiting ROS production and obstructing the activation of the MAPK/NF-κB pathway, thereby inhibiting the synthesis of inflammatory cytokines (Deng et al., 2022; Lee & Lee., 2022; Lee et al., 2009b; Lin et al., 2018; Radnai et al., 2009; Sul & Ra, 2021; Wang et al., 2020b). Quercitrin, *p*-hydroxybenzaldehyde, fraxetin, and vanillin relieved colitis *via* inhibiting the NF-κB pathway (Camuesco et al., 2004; Liu et al., 2024; Sun et al., 2024; Wu et al., 2009). Thence, ‘Fengtang’ plum seed EE mitigated colitis by suppressing the ROS/MAPK/NF-κB pathway, which may be attributed to these phenols and flavonoids.

In summary, our results showed that ‘Fengtang’ plum seed was abundant in phenolic and flavonoid compounds. ‘Fengtang’ plum seed EE lessened the inflammatory factor production by blocking the ROS/MAPK/NF-κB pathway in RAW264.7 cells induced by LPS. Furthermore, in DSS-induced colitis, ‘Fengtang’ plum seed EE significantly alleviated colitis by reducing inflammatory cytokine levels and ROS production through reducing levels of and enhancing the activities of CAT and SOD and reducing MDA content in tissue fluid and serum. Therefore, ‘Fengtang’ plum seed has great development value in functional foods.

Further research on activity-guided isolation is needed to determine which specific compounds are primarily responsible for the anti-inflammatory and anti-colitis effects of ‘Fengtang’ plum seed EE. Additionally, to support its application in functional foods, future work on safety evaluation, dose-response relationship, gut microbiota-related effect, bioavailability, and toxicity should be conducted to ensure the efficacy and safety of ‘Fengtang’ plum seed EE.

## 5 Conclusions

To our knowledge, this study first reported the anti-colitis effect and related mechanisms of ‘Fengtang’ plum seed. ‘Fengtang’ plum seed was abundant in phenolic and flavonoid compounds. A total of fifty-five compounds were identified using UHPLC-Q-Orbitrap-MS, comprising 16 phenolic components and 9 flavonoids. ‘Fengtang’ plum seed WE and EE exhibited moderate DPPH and ABTS scavenging effects. In LPS-induced RAW264.7, ‘Fengtang’ plum seed EE suppressed inflammatory factor release by blocking the ROS/MAPK/NF-κB pathway. Furthermore, it ameliorated DSS-induced colitis by diminishing the inflammatory cytokine levels and ROS production in colonic tissue fluid and serum. Hence, ‘Fengtang’ plum seed has great potentiality for exploitation in functional foods.

## CRediT authorship contribution statement

**Yuanquan Ran:** Writing – original draft, Methodology, Investigation. **Lu Jin:** Writing – original draft, Methodology, Investigation. **Furong Ding:** Methodology, Investigation. **Dan Long:** Resources, Investigation. **Guo Chen:** Validation, Resources. **Qiong Hu:** Validation, Supervision. **Bing Yang:** Resources. **Wenyu Wu:** Resources. **Dongxin Tang:** Writing – review & editing, Funding acquisition, Conceptualization. **Minyi Tian:** Writing – review & editing, Writing – original draft, Validation, Supervision, Methodology, Funding acquisition.

## Declaration of competing interest

The authors declare that they have no known competing financial interests or personal relationships that could have appeared to influence the work reported in this paper.

## Data Availability

Data will be made available on request.

## Appendix A Supplementary data

Supplementary data Image 1
