# Supplementary material for: ‘Fengtang’ plum seed waste: Phytochemicals and anti-inflammatory effects in vivo and in vitro
Source: Food Chem X. 2026 Mar 26;35:103793. doi: 10.1016/j.fochx.2026.103793 (PMC13054065; doi:10.1016/j.fochx.2026.103793)
Supplement: Supplementary file 1 — Supplementary data [file mmc1.docx]

**Supplementary Material**

Fig. S**1**. TPC, TFC, and radical scavenging action of ‘Fengtang’ plum seed WE and EE. (A) The total amounts of phenolic and ﬂavonoid in ‘Fengtang’ plum seed WE and EE. (B) ABTS radical scavenging activity of ‘Fengtang’ plum seed WE and EE, with BHT and ascorbic acid as positive controls. (C) DPPH radical scavenging activity of ‘Fengtang’ plum seed WE and EE, with BHT and ascorbic acid as positive controls. IC_50_: the concentration of the sample that scavenges 50% of the free radicals. ****p* < 0.001.

**Fig. S2.** UHPLC-Q-Orbitrap-MS chromatograms of ‘Fengtang’ plum seed WE and EE. (A) WE in positive ion mode; (B) WE in negative ion mode; (C) EE in positive ion mode; (D) EE in negative ion mode.

**Fig. S3.** (A) Eighteen phenolic compounds identified in ‘Fengtang’ plum seed WE and EE; (B) Eight flavonoids identified in ‘Fengtang’ plum seed WE and EE.

**Table S1**

Sequences of primers in qRT-PCR

| **Gene** | **Forward (5**′**-3**′**)** | **Reverse (5**′**-3**′**)** |
| --- | --- | --- |
| iNOS | TCGGGTTGAAGTGGTATGC | TCGGGTTGAAGTGGTATGC |
| COX-2 | TGACTGCCCAACTCCCAT | GAACCCAGGTCCTCGCTT |
| IL-1β | CCTGTGTCTTTCCCGTGGAC | CATCTCGGAGCCTGTAGTGC |
| TNF-α | GCAAAGGGAGAGTGGTCA | CTGGCTCTGTGAGGAAGG |
| IL-6 | ATTTCCTCTGGTCTTCTGG | GACTCTGGCTTTGTCTTTC |
| GAPDH | AGCCTCGTCCCGTAGACAAAA | GATGGCAACAATCTCCACTTT |

**Table S2**

Mouse weight loss, stool consistency, and blood in the stool scoring

| **Weight Loss** | **Score** | **Stool Consistency** | **Score** | **Blood in the Stool** | **Score** |
| --- | --- | --- | --- | --- | --- |
| 1-5 % | 1 | Normal | 0 | No blood | 0 |
| 5-10 % | 2 | Slight | 1 | Mild bleedin | 1 |
| 10-20 % | 3 | Loose | 2 | Moderate bleeding | 2 |
| > 20 % | 4 | Diarrhea | 3 | Severe bleeding | 3 |

**Table S3**

Molecular docking binding affinity of ligand with receptor (kcal/mol)

| **Receptor** | **PDB ID** | **Ligand** | **Binding affinity (kcal/mol)** |
| --- | --- | --- | --- |
| TNF-α | 7JRA | 2-Isopropylmalic acid | -5.44 |
|  | 7JRA | Leucic acid | -5.74 |
|  | 7JRA | Methyl vanillate | -5.8 |
|  | 7JRA | 4-Methoxyphenylacetic acid | -5.81 |
|  | 7JRA | Evodiamine | -7.44 |
|  | 7OM5 | 2-Isopropylmalic acid | -5.98 |
| EGFR | 7OM5 | 4-Methoxyphenylacetic acid | -6.21 |
|  | 7OM5 | Evodiamine | -6.92 |
| SRC | 3D7U | Evodiamine | -5.14 |
| HIF1 | 8HE0 | Evodiamine | -6 |
| PTGS2 (COX-2) | 6COX | Evodiamine | -5.63 |
| HSP90 | 2QG2 | Evodiamine | -7.87 |
| STAT3 | 6HJS | Evodiamine | -5.87 |
| TLR4 | 2Z62 | Evodiamine | -7.46 |

[1] 2-Hydroxy-4-methoxybenzaldehyde (Ding et al., 2019)

[2] Quinic acid (Deshpande et al., 2016; Masike et al., 2017)

[3] D-Gluconic acid (Yang et al., 2014)

[4] Sucrose (Valgimigli et al., 2012)

[5] Mannitol (Huang et al., 2020)

[6] Citric acid (Ledesma-Escobar et al., 2015; Wang et al., 2019)

[7] Scopolin (Cao et al., 2022)

[8] L-Phenylalanine (Scheubert et al., 2013)

[9] 6-Hydroxyindole

[10] 5-Hydroxymethylfurfural (Ran et al., 2024)

[11] L-Tryptophan (Ayre et al., 1994; Zhang et al., 2019)

[12] Clareolide

[13] 3-Furfuryl 2-pyrrolecarboxylate

[14] Protocatechuic acid (Gutierrez-Zetina et al., 2019)

[15] Gentisic acid (Ma et al., 2022; Cao et al., 2022)

[16] Vanillin (Hertzog et al., 2018; Wu et al., 2022)

[17] Ethyl ferulate (Jarrell et al., 2014)

[18] Orsellinic acid (Ma et al., 2016)

[19] o-Veratraldehyde (Ding et al., 2019; Wang et al., 2021a)

[20] 2-Isopropylmalic acid (Ricciutelli et al., 2019)

[21] Anisic aldehyde (Hertzog et al., 2018)

[22] Leucinic acid (Park et al., 2017)

[23] Aucubin (Gao et al., 2024)

[24] Prim-O-glucosylcimifugin (Zhang et al., 2021)

[25] Retrochalcone (Mittal & Kakkar, 2021)

[26] 3,5-Dimethoxy-4-hydroxybenzaldehyde (Wu et al., 2023a)

[27] Methyl vanillate (Lanzafame et al., 2017)

[28] Kaempferol-3-O-rutinoside (Wang et al., 2021b)

[29] 4-Methoxyphenylacetic acid

[30] Scopoletin (Cao et al., 2022; Shahzad et al., 2022; Zeng et al., 2015)

[31] Medicarpin (Taneja et al., 2020; Wang et al., 2019; Zhang et al., 2022)

[32] Procyanidin A2 (Lv et al., 2022; Wong-Paz et al., 2021)

[33] Ferulaldehyde (Wu et al., 2023b)

[34] Sauchinone (Niu et al., 2024)

[35] Sinapyl aldehyde (Silva et al., 2023)

[36] Isofraxidin (Liu et al., 2022; Majnooni et al., 2020)

[37] Ethyl 3,4-dihydroxybenzoate (Sinosaki et al., 2020)

[38] Quercitrin (Da Costa et al., 2016; Li et al., 2018)

[39] Azelaic acid (Abu-Reidah et al., 2013)

[40] Engeletin (Scigelova et al., 2021; Ye et al., 2021)

[41] Aurantio-obtusin (Qin et al., 2021; Xu et al., 2021)

[42] Fraxetin (Majnooni et al., 2020)

[43] β-Asarone (Sun et al., 2015)

[44] p-Hydroxybenzaldehyde (Ding et al., 2019; Ledesma-Escobar et al., 2015)

[45] Hesperetin (Jiao et al., 2020; Khan et al., 2022; Xu et al., 2009)

[46] 6-Gingerol (Asamenew et al., 2019; Zhao et al., 2025)

[47] Evodiamine (Xu et al., 2016; Zhang et al., 2018)

[48] Senkyunolide A (Liu et al., 2019; Lv et al., 2023)

[49] Methyl hexadecanoate (Nurdin et al., 2017)

[50] Germacrone (Ying et al., 2021)

[51] α-Linolenic acid (Aparamarta et al., 2018; Hejazi et al., 2009; Jabbar et al., 2009)

**Reference**

Abu-Reidah, I. M., Contreras, M. M., Arráez-Román, D., Segura-Carretero, A., & Fernández-Gutiérrez, A. (2013). Reversed-phase ultra-high-performance liquid chromatography coupled to electrospray ionization-quadrupole-time-of-flight mass spectrometry as a powerful tool for metabolic profiling of vegetables: *Lactuca sativa* as an example of its application.  *Journal of Chromatography. A*, *1313*, 212–227. https://doi.org/10.1016/j.chroma.2013.07.020.

Aparamarta, H. W., Qadariyah, L., Gunawan, S., & Ju, Y. H. (2018). Separation and identification of fatty acid in triacylglycerol isolated from *Calophyllum inophyllum* oil. *ARPN Journal of Engineering and Applied Sciences*, *3* (2), 442-451.

Asamenew, G., Kim, H. W., Lee, M. K., Lee, S. H., Kim, Y. J., Cha, Y. S., Yoo, S. M., & Kim, J. B. (2019). Characterization of phenolic compounds from normal ginger (*Zingiber officinale* Rosc.) and black ginger (*Kaempferia parviflora* Wall.) using UPLC–DAD–QToF–MS. *European Food Research and Technology*, *245*, 653–665. https://doi.org/10.1007/s00217-018-3188-z.

Ayre, C. R., Moro, L., & Becker, C. H. (1994). Effects of desorption method and photoionizing laser characteristics on molecular fragmentation. *Analytical Chemistry*, *66*, 1610–1619.

Cao, S., Hu, M., Yang, L., Li, M., Shi, Z., Cheng, W., Zhang, Y., Chen, F., Wang, S., & Zhang, Q. (2022). Chemical Constituent Analysis of *Ranunculus sceleratus* L. Using Ultra-High-Performance Liquid Chromatography Coupled with Quadrupole-Orbitrap High-Resolution Mass Spectrometry. *Molecules*, *27*, 3299. https://doi.org/10.3390/molecules27103299.

Da Costa, M. F., Galaverna, R. S., Pudenzi, M. A., Ruiz, A. L. T. G., De Carvalho, J. E., Eberlin, M. N., & Dos Santos, C. (2016). Profiles of phenolic compounds by FT-ICR MS and antioxidative and antiproliferative activities of *Stryphnodendron obovatum* Benth leaf extracts. *Analytical Methods*, *8*, 6056–6063. https://doi.org/10.1039/c6ay01272h.

Deshpande, S., Matei, M. F., Jaiswal, R., Bassil, B. S., Kortz, U., & Kuhnert, N. (2016). Synthesis, Structure, and Tandem Mass Spectrometric Characterization of the Diastereomers of Quinic Acid. *Journal of Agricultural and Food Chemistry*, *64*, 7298–7306. https://doi.org/10.1021/acs.jafc.6b02472.

Ding, F., Liu, J., Du, R., Yu, Q., Gong, L., Jiang, H., & Rong, R. (2019). Qualitative and Quantitative Analysis for the Chemical Constituents of *Tetrastigma hemsleyanum* Diels et Gilg Using Ultra-High Performance Liquid Chromatography/Hybrid Quadrupole-Orbitrap Mass Spectrometry and Preliminary Screening for Anti-Influenza Virus Components. *Evidence-based Complementary and Alternative Medicine*, *2019*, 9414926. https://doi.org/10.1155/2019/9414926.

Gao, X., Gao, F., Pan, X., Wang, Y., Liu, X., Li, Y., Nan, G., Bai, L., & Sun, W. (2024). Optimization of extracting technology of iridoid glycosides and phenylethanoid glycosides from *Pedicularis decora* Franch. by G1-entropy-coupled response surface methodology and rapid identification by UPLC-Q/TOF-MS. *Journal of Liquid Chromatography & Related TeChnologies*, *47*, 132–144. https://doi.org/10.1080/10826076.2024.2333518.

Gutierrez-Zetina, S. M., Gonzalez-Manzano, S., Perez-Alonso, J. J., Gonzalez-Paramas, A. M., & Santos-Buelga, C. (2019). Preparation and Characterization of Protocatechuic Acid Sulfates. *Molecules*, *24*, 307. https://doi.org/10.3390/molecules24020307.

Hejazi, L., Ebrahimi, D., Guilhaus, M., & Hibbert, D. B. (2009). Discrimination among geometrical isomers of α-linolenic acid methyl ester using low energy electron ionization mass spectrometry. *Journal of the American Society for Mass Spectrometry*, *20*, 1272–1280. https://doi.org/10.1016/j.jasms.2009.02.027.

Hertzog, J., Carré, V., Dufour, A., & Aubriet, F. (2018). Semi-Targeted Analysis of Complex Matrices by ESI FT-ICR MS or How an Experimental Bias may be Used as an Analytical Tool. *Journal of the American Society for Mass Spectrometry*, *29*, 543–557. https://doi.org/10.1007/s13361-017-1865-y.

Huang, G., Liang, J., Chen, X., Lin, J., Wei, J., Huang, D., Zhou, Y., Sun, Z., & Zhao, L. (2020). Isolation and Identification of Chemical Constituents from Zhideke Granules by Ultra-Performance Liquid Chromatography Coupled with Mass Spectrometry. *Journal of Analytical Methods in Chemistry*, *2020*, 8889607. https://doi.org/10.1155/2020/8889607.

Jabbar, A., Ali, A., Tawab, A., Haque, A., & Iqbal, M. (2014). Fatty acid profiling of lipid A isolated from indigenous *Salmonella* Typhi strain by gas chromatography mass spectrometry. *Journal of the Chemical Society of Pakistan*, *36* (1), 140–149.

Jarrell, T. M., Marcum, C. L., Sheng, H., Owen, B. C., O'Lenick, C. J., Maraun, H., Bozell, J. J., & Kenttämaa, H. I. (2014). Characterization of organosolv switchgrass lignin by using high performance liquid chromatography/high resolution tandem mass spectrometry using hydroxide-doped negative-ion mode electrospray ionization. *Green Chemistry*, *16*, 2713–2727. https://doi.org/10.1039/C3GC42355G.

Jiao, Q., Xu, L., Jiang, L., Jiang, Y., Zhang, J., & Liu, B. (2020). Metabolism study of hesperetin and hesperidin in rats by UHPLC-LTQ-Orbitrap MS^n^. *Xenobiotica*, *50*, 1311–1322. https://doi.org/10.1080/00498254.2019.1567956.

Khan, M., Rauf, W., Habib, F. E., Rahman, M., Iqbal, S., Shehzad, A., & Iqbal, M. (2022). Hesperidin identified from *Citrus* extracts potently inhibits HCV genotype 3a NS3 protease. *BMC Complementary Medicine and Therapies*, *22*, 98. https://doi.org/10.1186/s12906-022-03578-1.

Lanzafame, G. M., Sarakha, M., Fabbri, D., & Vione, D. (2017). Degradation of Methyl 2-Aminobenzoate (Methyl Anthranilate) by H_2_O_2_/UV: Effect of Inorganic Anions and Derived Radicals. *Molecules,* *22*, 619. https://doi.org/10.3390/molecules22040619.

Ledesma-Escobar, C. A., Priego-Capote, F., & Luque de Castro, M. D. (2015). Characterization of lemon (*Citrus limon*) polar extract by liquid chromatography-tandem mass spectrometry in high resolution mode.  *Journal of Mass Spectrometry*, *50*, 1196–1205. https://doi.org/10.1002/jms.3637.

Li, A., Hou, X., & Wei, Y. (2018). Fast screening of flavonoids from switchgrass and *Mikania micrantha* by liquid chromatography hybrid-ion trap time-of-flight mass spectrometry. *Analytical Methods*, *10*, 109–122. https://doi.org/10.1039/c7ay02103h.

Liu, X., Chen, H., Su, G., Song, P., Jiang, M., & Gong, J. (2019). An animal research and a chemical composition analysis of a Chinese prescription for pulmonary fibrosis: Yangfei Huoxue Decoction. *Journal of Ethnopharmacology*, *245*, 112126. https://doi.org/10.1016/j.jep.2019.112126.

Liu, H., Yang, L., Wan, C., Li, Z., Yan, G., Han, Y., Sun, H., & Wang, X. (2022). Exploring potential mechanism of ciwujia tablets for insomnia by UPLC-Q-TOF-MS/MS, network pharmacology, and experimental validation. *Frontiers in Pharmacology*, *13*, 990996. https://doi.org/10.3389/fphar.2022.990996.

Liu, R., Sun, Y., Di, D., Zhang, X., Zhu, B., & Wu, H. (2023). PI3K/AKT/SERBP-1 pathway regulates *Alisma orientalis* beverage treatment of atherosclerosis in APOE-/- high-fat diet mice. *Pharmaceutical Biology*, *61*, 473–487. https://doi.org/10.1080/13880209.2023.2168020.

Lv, M., Wang, Y., Wan, X., Han, B., Yu, W., Liang, Q., Xiang, J., Wang, Z., Liu, Y., Qian, Y., & Xu, F. (2022). Rapid Screening of Proanthocyanidins from the Roots of *Ephedra sinica* Stapf and its Preventative Effects on Dextran-Sulfate-Sodium-Induced Ulcerative Colitis. *Metabolites*, *12*, 957. https://doi.org/10.3390/metabo12100957.

Lv, Y., Xu, X., Yang, J., Gao, Y., Xin, J., Chen, W., Zhang, L., Li, J., Wang, J., Wei, Y., Wei, X., He, J., & Zu, X. (2023). Identification of chemical components and rat serum metabolites in Danggui Buxue decoction based on UPLC-Q-TOF-MS, the UNIFI platform and molecular networks. *RSC Advances*, *13*, 32778–32785. https://doi.org/10.1039/d3ra04419j.

Ma, Y., Tian, T., Xie, W., Jin, Y., Xu, H., Zhang, L., & Du, Y. (2016). Major phenolic acids in *Usneae Filum* by UHPLC-Triple-TOF-MS. *Chinese Traditional and Herbal Drugs,* *47*, 392–400. https://doi.org/10.7501/j.issn.0253-2670.2016.03.007.

Ma, Y., Wang, C., Wang, F., Wang, M., Tian, We., Wu, L., & Niu, L. (2022). Rapid identification of chemical components of *Folium Photiniae* based on UPLC-Q-TOF-MS. *Chinese Traditional and Herbal Drugs,* *53*, 6401–6411. https://doi.org/10.7501/j.issn.0253-2670.2022.20.012.

Majnooni, M. B., Fakhri, S., Shokoohinia, Y., Mojarrab, M., Kazemi-Afrakoti, S., & Farzaei, M. H. (2020). Isofraxidin: Synthesis, Biosynthesis, Isolation, Pharmacokinetic and Pharmacological Properties. *Molecules, 25*, 2040. https://doi.org/10.3390/molecules25092040.

Masike, K., Mhlongo, M. I., Mudau, S. P., Nobela, O., Ncube, E. N., Tugizimana, F., George, M. J., & Madala, N. E. (2017). Highlighting mass spectrometric fragmentation differences and similarities between hydroxycinnamoyl-quinic acids and hydroxycinnamoyl-isocitric acids. *Chemistry Central Journal*, *11*, 29. https://doi.org/10.1186/s13065-017-0262-8.

Mittal, A., & Kakkar, R. (2021). Synthetic methods and biological applications of retrochalcones isolated from the root of *Glycyrrhiza* species: A review. *Results in Chemistry*, *3*, 100216. https://doi.org/10.1016/j.rechem.2021.100216.

Niu, J., Jia, X., Yang, N., Ran, Y., Wu, X., Ding, F., Tang, D., & Tian, M. (2024). Phytochemical analysis and anticancer effect of *Camellia oleifera* bud ethanol extract in non-small cell lung cancer A549 cells. *Frontiers in Pharmacology*, *15*, 1359632. https://doi.org/10.3389/fphar.2024.1359632.

Nurdin, M., Fatma, F., Natsir, M., & Wibowo, D. (2017). Characterization of methyl ester compound of biodiesel from industrial liquid waste of crude palm oil processing. *Analytical Chemistry Research*, *12*, 1–9. http://creativecommons.org/licenses/by-nc-nd/4.0/.

Park, B., Hwang, H., Chang, J. Y., Hong, S. W., Lee, S. H., Jung, M. Y., Sohn, S. O., Park, H. W., & Lee, J. H. (2017). Identification of 2-hydroxyisocaproic acid production in lactic acid bacteria and evaluation of microbial dynamics during kimchi ripening. *Scientific Reports*, *7*, 10904. https://doi.org/10.1038/s41598-017-10948-0.

Qin, S. H., Xu, Y., Li, K. I., Gong, K. Y., Peng, J., Shi, S. I., Yan, F., & Cai, W. (2021). Identification of Metabolites of Aurantio-Obtusin in Rats Using Ultra-High-Performance Liquid Chromatography-Q-Exactive Orbitrap Mass Spectrometry with Parallel Reaction Monitoring. *Journal of Analytical Methods in Chemistry*, *2021*, 6630604. https://doi.org/10.1155/2021/6630604.

Ran, Y., Yang, L., Jia, X., Zhao, H., Hu, Q., Yang, B., Tang, D., & Tian, M. (2024). Phytochemical composition and anticancer effect of *Akebia trifoliata* seed in non-small cell lung cancer A549 cells. *Arabian Journal of Chemistry*, *17*, 106020. https://doi.org/10.1016/j.arabjc.2024.106020.

Ricciutelli, M., Moretti, S., Galarini, R., Sagratini, G., Mari, M., Lucarini, S., Vittori, S., & Caprioli, G. (2019). Identification and quantification of new isomers of isopropyl-malic acid in wine by LC-IT and LC-Q-Orbitrap. *Food Chemistry*, *294*, 390–396. https://doi.org/10.1016/j.foodchem.2019.05.068.

Scheubert, K., Hufsky, F., & Böcker, S. (2013). Computational mass spectrometry for small molecules. *Journal of Cheminformatics*, *5*, 12. https://doi.org/10.1186/1758-2946-5-12.

Scigelova, M., Hornshaw, M., Giannakopulos, A., & Makarov, A. (2011). Fourier transform mass spectrometry. *Molecular & Cellular Proteomics*, *10*, 1–19. https://doi.org/10.1074/mcp.M111.009431.

Shahzad, M. N., Ahmad, S., Tousif, M. I., Ahmad, I., Rao, H., Ahmad, B., & Basit, A. (2022). Profiling of phytochemicals from aerial parts of *Terminalia neotaliala* using LC-ESI-MS2 and determination of antioxidant and enzyme inhibition activities. *PloS One*, *17*, e0266094. https://doi.org/10.1371/journal.pone.0266094.

Silva, D., Sousa, A. C., Robalo, M. P., & Martins, L. O. (2023). A wide array of lignin-related phenolics are oxidized by an evolved bacterial dye-decolourising peroxidase. *New Biotechnology*, *77*, 176–184. https://doi.org/10.1016/j.nbt.2022.12.003.

Sinosaki, N. B. M., Tonin, A. P. P., Ribeiro, M. A. S., Poliseli, C. B., Roberto, S. B., Silveira, R. D., Visentainer, J. V., Santos, O. O., & Meurer, E. C. (2020). Structural study of phenolic acids by triple quadrupole mass spectrometry with electrospray ionization in negative mode and H/D isotopic exchange. *Journal of the Brazilian Chemical Society*, *31*, 402–408. http://dx.doi.org/10.21577/0103-5053.20190197.

Sun, H., Liu, C., Zhang, A. H., Han, Y., Yan, G. L., Wang, P., & Wang, X. J. (2015). Rapid discovery and global characterization of multiple constituents from Kai-Xin-San using an integrated MS^E^ data acquisition mode strategy based on ultra-performance liquid chromatography coupled to electrospray ionization/quadrupole-time-of-flight mass spectrometry. *Analytical Methods*, *7*, 279–286. https://doi.org/10.1039/c4ay01954g.

Taneja, I., Raghuvanshi, A., Rama Raju, K. S., Awasthi, P., Rashid, M., Singh, S., Goel, A., Singh, S. P., & Wahajuddin, M. (2020). Bioavailability, tissue distribution and excretion studies of a potential anti-osteoporotic agent, medicarpin, in female rats using validated LC-MS/MS method. *Journal of Pharmaceutical and Biomedical Analysis*, *180*, 112978. https://doi.org/10.1016/j.jpba.2019.112978.

Valgimigli, L., Gabbanini, S., & Matera, R. (2012). Analysis of maltose and lactose by U-HPLC-ESI-MS/MS. In: Preedy, V.R. (Ed.), Dietary Sugars: Chemistry, Analysis, Function and Effects. *Food and Nutritional Components in Focus*. The Royal Society of Chemistry*,* pp. 443–463. https://doi.org/10.1039/9781849734929-00443.

Wang, Z., Liu, J., Zhong, X., Li, J., Wang, X., Ji, L., & Shang, X. (2019). Rapid Characterization of Chemical Components in Edible Mushroom *Sparassis crispa* by UPLC-Orbitrap MS Analysis and Potential Inhibitory Effects on Allergic Rhinitis. *Molecules,* *24*, 3014. https://doi.org/10.3390/molecules24163014.

Wang, K., Tian, J., Li, Y., Liu, M., Chao, Y., Cai, Y., Zheng, G., & Fang, Y. (2021a). Identification of Components in Citri Sarcodactylis Fructus from Different Origins via UPLC-Q-Exactive Orbitrap/MS. *ACS Omega*, 6, 17045–17057. https://doi.org/10.1021/acsomega.1c02124.

Wang, Q., Zou, Z., Zhang, Y., Lin, P., Lan, T., Qin, Z., Xu, D., Wu, H., & Yao, Z. (2021b). Characterization of chemical profile and quantification of major representative components of *Wendan* decoction, a classical traditional Chinese medicine formula. *Journal of Separation Science*, *44*, 1036–1061. https://doi.org/10.1002/jssc.202000952.

Wong-Paz, J. E., Guyot, S., Aguilar-Zárate, P., Muñiz-Márquez, D. B., Contreras-Esquivel, J. C., & Aguilar, C. N., (2021). Structural characterization of native and oxidized procyanidins (condensed tannins) from coffee pulp (*Coffea arabica*) using phloroglucinolysis and thioglycolysis-HPLC-ESI-MS. *Food Chemistry*, *340*, 127830. https://doi.org/10.1016/j.foodchem.2020.127830.

Wu, X., Pan, Z., Bjelić, S., Hemberger, P., & Bodi, A. (2022). Unimolecular thermal decarbonylation of vanillin stifled by the bimolecular reactivity of methyl-loss intermediate. *Journal of Analytical and Applied Pyrolysis*, *161*, 105410. https://doi.org/10.1016/j.jaap.2021.105410.

Wu, N., Tunnisaguli, A., Chang, J., & Li, G. (2023a). Screening of anti-asthmatic active extracts from *Sorbus tianschanica* Rupr. and component analysis. *Central South Pharmacy, 21*, 863–869. https://doi.org/10.7539/j.issn.1672-2981.2023.04.005.

Wu, X., Wei, F., Ding, F., Yang, N., Niu, J., Ran, Y., & Tian, M., (2023b). Phytochemical analysis, antioxidant, antimicrobial, and anti-enzymatic properties of *Alpinia coriandriodora* (sweet ginger) rhizome. *Frontiers in Plant Science, 14*, 1284931. https://doi.org/10.3389/fpls.2023.1284931

Xu, F., Liu, Y., Zhang, Z., Yang, C., & Tian, Y. (2009). Quasi-MSn identification of flavanone 7-glycoside isomers in *Da Chengqi Tang* by high performance liquid chromatography-tandem mass spectrometry. *Chinese Medicine*, *4*, 15. https://doi.org/10.1186/1749-8546-4-15.

Xu, H., Niu, H., He, B., Cui, C., Li, Q., & Bi, K. (2016). Comprehensive Qualitative Ingredient Profiling of Chinese Herbal Formula Wu-Zhu-Yu Decoction via a Mass Defect and Fragment Filtering Approach Using High Resolution Mass Spectrometry. *Molecules, 21*, 664. https://doi.org/10.3390/molecules21050664.

Xu, L., Zhang, Z., Hao, F., Zhou, W., Tang, X., & Gao, Y. (2021). A comparative study of aurantio-obtusin metabolism in normal and liver-injured rats by ultra performance liquid chromatography quadrupole time-of-flight mass spectrometry. *Journal of Pharmaceutical and Biomedical Analysis*, *196*, 113896. https://doi.org/10.1016/j.jpba.2021.113896.

Yang, H., Lin, W., Zhang, J., Lin, W., Xu, P., Li, J., & Ling, X., (2014). Metabonomic analysis of the toxic effects of TM208 in rat urine by HPLC-ESI-IT-TOF/MS. *Journal of Chromatography B,* *959*, 49–54. https://doi.org/10.1016/j.jchromb.2014.03.036.

Ye, X., Wu, J., Yang, J., Kantawong, F., Kumsaiyai, W., & Zeng, J. (2021). Research progress on chemical constituents of *Gynura divaricate* and mass spectrometry-based fragmentation rules of representative components. *Chinese Traditional and Herbal Drugs,* *52*, 6687–6700. https://doi.org/10.7501/j.issn.0253-2670.2021.21.028.

Ying, Y., Yu, M., Xiao, J., & Shen, Q. (2021). Metabolites and Metabolic Pathway Analysis of Germacrone in Rats by UHPLC-Q-Orbitrap HRMS. *Chinese Journal of Modern Applied Pharmacy, 38,* 430–438. https://doi.org/10.13748/j.cnki.issn1007-7693.2021.04.009.

Zeng, Y., Li, S., Wang, X., Gong, T., Sun, X., & Zhang, Z. (2015). Validated LC-MS/MS Method for the Determination of Scopoletin in Rat Plasma and Its Application to Pharmacokinetic Studies. *Molecules, 20*, 18988–19001. https://doi.org/10.3390/molecules201018988.

Zhang, Z., Fang, T., Zhou, H., Yuan, J., & Liu, Q. (2018). Characterization of the *in Vitro* Metabolic Profile of Evodiamine in Human Liver Microsomes and Hepatocytes by UHPLC-Q Exactive Mass Spectrometer. *Frontiers in Pharmacology*, *9*, 130. https://doi.org/10.3389/fphar.2018.00130

Zhang, P., Chan, W., Ang, I. L., Wei, R., Lam, M. M. T., Lei, K. M. K., & Poon, T. C. W. (2019). Revisiting Fragmentation Reactions of Protonated α-Amino Acids by High-Resolution Electrospray Ionization Tandem Mass Spectrometry with Collision-Induced Dissociation. *Scientific Reports*, *9*, 6453. https://doi.org/10.1038/s41598-019-42777-8.

Zhang, F. X., Yuan, Y. L. L., Cui, S. S., Wang, G. H., & Li, R. M. (2021). Revealing the potential pharmacological mechanism of traditional Chinese medicine by integrating metabolite profiling of a Q-marker and network pharmacology, prim-*O*-glucosylcimifugin as an example. *New Journal of Chemistry*, *45* (34), 15571–15581. https://doi.org/10.1039/d1nj02209a.

Zhang, J., Wang, J., Wang, Y., Chen, M., Shi, X., Zhou, X., & Zhang, Z. (2022). Phytochemistry and Antioxidant Activities of the Rhizome and Radix of *Millettia speciosa* Based on UHPLC-Q-Exactive Orbitrap-MS. *Molecules*, *27*, 7398. https://doi.org/10.3390/molecules27217398.

Zhao, H., Wang, Q., Yang, L., Ran, Y., Hu, Q., Hong, Y., & Tian, M. (2025). Phytochemical analysis, antioxidant, anti-inflammatory and enzyme inhibitory activities of bean pear (*Pyrus calleryana fruit*). *Frontiers in plant science, 16*, 1521990. https://doi.org/10.3389/fpls.2025.1521990.
